# Comparative genomics reveals new functional insights in uncultured MAST species

**DOI:** 10.1038/s41396-020-00885-8

**Published:** 2021-01-15

**Authors:** Aurelie Labarre, David López-Escardó, Francisco Latorre, Guy Leonard, François Bucchini, Aleix Obiol, Corinne Cruaud, Michael E. Sieracki, Olivier Jaillon, Patrick Wincker, Klaas Vandepoele, Ramiro Logares, Ramon Massana

**Affiliations:** 1 Department of Marine Biology and Oceanography, Institut de Ciències del Mar (CSIC), Barcelona, Catalonia Spain; 2 Department of Zoology, University of Oxford, Oxford, UK; 3 Department of Plant Biotechnology and Bioinformatics, Ghent University, Technologiepark, Ghent, Belgium; 4 VIB Center for Plant Systems Biology, Technologiepark, Ghent, Belgium; 5 Commissariat à l’Energie Atomique et aux Energies Alternatives (CEA), Institut de biologie François-Jacob, Genoscope, Evry, France; 6 National Science Foundation, Alexandria, VA USA; 7 Metabolic Genomics, Institut de Biologie François Jacob, Genoscope, CEA, CNRS, Univ Evry, Université Paris Saclay, 91000 Evry, France; 8 Research Federation for the study of Global Ocean Systems Ecology and Evolution, Ghent, Belgium; 9 Bioinformatics Institute Ghent, Ghent University, 9052 Paris, France

**Keywords:** Genomics, Microbiology

## Abstract

Heterotrophic lineages of stramenopiles exhibit enormous diversity in morphology, lifestyle, and habitat. Among them, the marine stramenopiles (MASTs) represent numerous independent lineages that are only known from environmental sequences retrieved from marine samples. The core energy metabolism characterizing these unicellular eukaryotes is poorly understood. Here, we used single-cell genomics to retrieve, annotate, and compare the genomes of 15 MAST species, obtained by coassembling sequences from 140 individual cells sampled from the marine surface plankton. Functional annotations from their gene repertoires are compatible with all of them being phagocytotic. The unique presence of rhodopsin genes in MAST species, together with their widespread expression in oceanic waters, supports the idea that MASTs may be capable of using sunlight to thrive in the photic ocean. Additional subsets of genes used in phagocytosis, such as proton pumps for vacuole acidification and peptidases for prey digestion, did not reveal particular trends in MAST genomes as compared with nonphagocytotic stramenopiles, except a larger presence and diversity of V-PPase genes. Our analysis reflects the complexity of phagocytosis machinery in microbial eukaryotes, which contrasts with the well-defined set of genes for photosynthesis. These new genomic data provide the essential framework to study ecophysiology of uncultured species and to gain better understanding of the function of rhodopsins and related carotenoids in stramenopiles.

## Introduction

Oceans are the largest habitats on Earth, and living biomass in these systems is dominated by planktonic microbes [1]. Together, they introduce heterogeneity into the ocean, govern trophic interactions, and drive energy and nutrient flows [2]. Depending on the way microbes acquire energy and food, they stand along a trophic spectrum between phototrophs, which synthesize organic matter using solar energy and heterotrophs, which live at the expense of acquired organic matter. The study of trophic strategies is of primary interest to understand the ecological role and behavior of microbial species. This basic information is not always easy to access, especially because as seen in molecular surveys, the vast majority of microbial diversity has not been cultured and therefore remains uncharacterized [3]. Within marine microbial eukaryotes, an important component of this unknown diversity are the marine stramenopiles (MASTs) lineages [4, 5], placed in different positions of the stramenopile radiation that include phototrophs, phagotrophs, mixotrophs, osmotrophs, and parasites [6, 7]. Currently, MASTs are divided into 18 phylogenetic clades [8], each one potentially harboring many species which are essentially uncultured, with only two exceptions, *Incisomonas marina* (MAST-3) and *Pseudophyllomitus vesiculosus* (MAST-6). A clear assignment of the trophic strategy of MASTs is also challenging because of their small size and lack of recognizable morphological features. Partial data exist for a few clades, some MAST-3 are parasites (for example, the diatom parasite *Solenicola setigera* belongs to this clade [9]), MAST-1 and MAST-4 are active bacterivores [10], but this elementary knowledge is still unknown for many other MAST lineages.

In the last years, MASTs have been under the hook with a few single-cell genomics (SCG) studies [11]. Despite inherent methodological limitations, such as uneven coverage, chimeric assemblies, and increased contamination [12], SCG is becoming widely used to access the genomes of uncultured microbial species [13, 14], therefore expanding our knowledge on marine microbial life and their metabolic potential. Recently, a catalog of more than 900 single amplified genomes (SAGs) isolated during the Tara Oceans expedition has been described based on their 18S rDNA genes [15]; many of them affiliated to diverse MAST clades and some were chosen for genome sequencing. In a first study, several MAST-4 SAGs were used to evaluate a computational solution to improve genome completeness by combining the sequencing reads of single cells into a coassembly [16]. Another study used this coassembling approach to obtain the genome of five MAST-3 and MAST-4 species and explore their functional ecology and oceanic distribution. This study revealed functional differences in the motility apparatus and feeding spectra, and the presence of rhodopsins in one species. Here, we extended the dataset to 15 MAST species using SAGs from Tara Oceans and from other projects. We investigated their trophic strategy using a comparative genomics model, and focused on a set of gene families relevant for phagocytosis.

Phagocytosis is a distinct form of endocytosis that incorporates particles > 0.45 µm in diameter through the formation of membrane-bound vesicles called phagosomes. After maturation, phagosomes fuse with lysosomes and become a final phagolysosome where prey cells are degraded [17, 18]. Lysosomes are important organelles that can contain more than 50 degradative enzymes (targeting proteins, carbohydrates, or nucleic acids) commonly named acid hydrolases as they are activated at acidic conditions (i.e., pH < 5). To maintain the acidic medium and keep control over the digestive enzymes, phagolysosomes accumulate H^+^ ions by the action of the vacuolar-type H^+^-translocating ATPase (V-ATPase) [19]. Other proton pumps such as the vacuolar-type H^+^-translocating pyrophosphatase (V-PPase) may also participate in acidification [20]. The two proton pumps obtain their energy by hydrolyzing phosphate bonds, in ATP or inorganic pyrophosphate, respectively, [21], and represent distinct classes of ion translocases with no sequence homology. Functional related genes that are gaining momentum in marine microbial ecology are the rhodopsins. Microbial type I rhodopsins are photoactive proteins containing a retinal chromophore that work as light-driven proton pumps or photoreceptors [22, 23]. They are widely present in marine microbes [24, 25] and have been found in MAST-4-C [26] and highly expressed in a growing MAST-4A population [27]. It has been suggested that besides energy processing, rhodopsins can participate in food vacuole acidification in eukaryotic phagotrophs [28].

In this study, we have analyzed the genomes of 140 single cells retrieved during the Tara Oceans expedition as well as at the Blanes Bay Microbial Observatory (BBMO). These cells affiliate within seven MAST clades highly represented in marine molecular surveys [6]. The 140 SAGs have been further coassembled into 15 genomes of relatively high quality and subsequently analyzed by comparative genomics together with other well-characterized stramenopiles. We first focused on assigning a trophic function to these uncultured clades by comparative genomics, and then analyzed the enrichment of the degradative enzymes peptidases according to trophic function. We also considered in detail the presence and diversity of proton pumps and microbial rhodopsins in MASTs to further understand the potential physiological cell capabilities and the role of light in phagolysosome acidification.

## Material and methods

### SAG sequencing, assembly, and coassembly

Epipelagic microbial communities sampled during the Tara Oceans expedition were used for flow cytometry cell sorting at the Single Cell Sorting Center in Bigelow (scgc.bigelow.org) based on size and the presence or absence of pigments. Whole-genome amplification from single cells was done with MDA, and SAGs were taxonomically classified by sequencing their 18S rDNA amplified with universal eukaryotic primers. Details of the methods used and a complete list of taxa ID for all SAGs collected in Tara are presented in Sieracki et al. [15]. Overall, 74 of the SAGs used here have been sequenced and analyzed previously [16, 26, 29], while 50 SAGs are new from this study (Table S1). We did a single-cell sorting effort at the BBMO in May 2018 using similar protocols that provided 16 additional SAGs. Sequencing libraries for cells collected in Tara were prepared as described before [26], while we used the KAPA or NextEra preparation kits in BBMO cells. SAGs were paired-end sequenced (reads of 110 bp in Tara and 250 bp in BBMO) in different Illumina platforms and sequencing services (Table S1).

After adapter trimming and cleaning of the raw reads using Trimmomatic v. 0.32 [30] (reads with a Phred score < 20 and <100 bp were discarded), we performed a digital k-mer-based normalization with BBNorm (sourceforge.net/projects/bbmap/) that reduces the average error rate and allows downsampling of reads for a better coverage distribution (a critical issue with MDA products). An initial de novo assembly using the De Bruijn graph assembler SPAdes [31], combining information from 21, 33, and 55 k-mer sizes, was generated for every individual SAG read set. Based on previous work [16, 29], we followed a stringent coassembly strategy. SAGs eligible for coassembly were those with very high 18S rDNA similarity (>99.5%) and average nucleotide identity (>95%), and tetranucleotide homogeneity verified with the Emergent Self-Organizing Maps tool (http://databionic-esom.sourceforge.net) using a 1 bp sliding window in fragmented contigs of 2.5–5 kb. The formed clusters were validated with robust estimates of mean and variance (“Robust ZT” option). Coassembly was done with SPAdes including the “single-cell” option. We identified (and later removed) prokaryotic contamination in the assembled scaffolds with the default parameters of EukRep [32] and BlobTools [33]. Contigs with divergent GC content values in each coassembly (outside the range of mean ± 10% standard deviation) were also removed. In one of the sequencing batches, cross-contamination between SAGs in the same Illumina lane occurred due to HiSeq reagents problems. We computed the average nucleotide identity [34] between contigs in all pairs of individual SAGs, identified problematic contigs (those that share similarity > 99% in fragments longer than 300 bp), and removed those from the SAG where they had the lowest k-mer read coverage. In the final coassemblies, contigs shorter than 1 kb were removed, and genome statistics were computed with QUAST [35]. Genome completeness was determined by the presence of 248 universal, single-copy core eukaryotic genes with CEGMA [36] or the presence of 303 single-copy eukaryotic orthologous genes with BUSCO v3 [37].

### Gene predictions, gene family inference, and functional annotation

Gene predictions from the coassembled genomes started by using the CEGMA and BUSCO retrieved genes to train SNAP (http://korflab.ucdavis.edu/software.html), which generates a set of ab initio gene models. In parallel, GENEMARK-ES [38] was run to obtain another set of predicted genes. Both sets were then used as input for a first run on the MAKER [39] pipeline. The candidate genes identified were then used as the training dataset input in a second run of MAKER, with default settings, to train the program AUGUSTUS [40], finally providing transcripts and protein predictions for each coassembled genome. The pipeline used can be found on GitHub (https://github.com/guyleonard/gene_prediction_pipeline).

Predicted coding sequences (CDS) from the coassembled MAST genomes were loaded into a custom instance of the PLAZA framework [41] together with the CDS of other stramenopiles and nonstramenopile model species (Fig. S1). Based on an “all-against-all” protein sequence similarity search done with DIAMOND v. 0.9.18 [42] (“more sensitive” mode with a maximum *e*-value cutoff of 10^−5^ and retaining up to 2500 hits), orthologous gene families were delineated with OrthoFinder v. 2.3.3 [43] (default parameters). Functional annotation of all CDS was performed using InterProScan v. 5.39–77.0 [44], including mapping InterPro entries to GO annotations. For the model organisms in the database (Fig. S1), GO annotations were retrieved from the GO website. Finally, functional enrichment analyses were performed to assign informative InterPro and GO terms to each orthologous gene family. The enrichment analysis used the hypergeometric distribution with a maximum Bonferroni corrected *p* value cutoff of 0.05, and all coding genes from the organisms included in the gene family as background frequency. Enriched functional annotations were retained when present in at least half of the genes in the family.

### Comparative genomics analysis

We used a computational model designed to predict, using genomic data, if an organism has the ability to be phagocytotic (able to capture prey), photosynthetic (able to fix inorganic carbon), or prototrophic (self-sufficient producer of essential amino acids or vitamins) [45]. The model is based on clusters of shared proteins among a large diversity of eukaryotic genomes and on an evaluation of their enrichment in organisms adopting different lifestyles. The presence of specific proteins in the query genomes, detected by a search with HMM models, is used to predict the lifestyle of unknown organisms.

On a second level, we used the number of copies for each orthologous gene family (or orthologous group, OG) in every species to identify broad patterns within the 30 stramenopile species. OGs found in only one species were discarded, and the number of genes per OGs was normalized to percentages in each genome. Based on the OG table, genomes were compared using Bray–Curtis dissimilarities and analyzed by nonmetric multidimensional scaling (NMDS) with the R package vegan v2.5–6 [46]. The grouping of species based on trophic lifestyle was tested by a PERMANOVA analysis using vegan’s function *adonis2()*. A multilevel pattern analysis to identify OGs that characterize a given trophic mode (indicator value (IndVal) > 0.7 and *p* value < 0.05) was performed using the function *multipatt()* implemented in the R package indicspecies v1.7.9 [47]. A heatmap displaying OGs annotated as peptidases and proteases was created with R package pheatmap v1.0.12 [48], using Ward’s method for hierarchical clustering with log_10_-transformed OGs gene counts (with a pseudocount of 1).

### Homology searches and phylogenetic analyses for specific proteins

Protein sequences from three gene families of proton pumps were retrieved from public databases. Reference sequences for V-ATPases were extracted from Mulkidjanian et al. [49], while for V-PPases we used the phylogenetic tree in Goodenough et al. [20]. Rhodopsin reference sequences were collected from several articles [28, 50, 51], and the MicRhoDE project [52]. Using these reference datasets, homologous MAST sequences were identified by sequence similarity using BLAST v.2.2.28 (maximum *e*-value threshold of 10^−5^). The selected contigs were checked to discard potential bacterial contamination. Homology searches using Pfam domains were conducted against the key enzymes involved in retinal formation: GGPP synthase (PF00348), phytoene synthase (PF00484.18), phytoene dehydrogenase (PF01493.23), lycopene cyclase (PF05834), and β-carotene 15,15′-dioxygenase (PF15461.5). Selected sequences were aligned with MAFFT v7.470 [53] (--globalpair) and trimmed with TRIMAL v1.4 [54] (-automated option) to obtain a curated subset for phylogenetic analyses. Phylogenetic trees were constructed with the maximum likelihood method using the LG+F+R6 substitution model in IQ-TREE [55] and topology support was determined with 1000 bootstrap replicates. Rhodopsin expression data were retrieved from the Marine Atlas of Tara Oceans Unigenes database (MATOU) [56], which presents expressed eukaryotic genes clustered at 95% identity. Rhodopsin MAST sequences were used as query in BLAST 2.7.1 against MATOU v1 and we kept the most similar unigene for each type.

## Results

### A new set of MAST genomes

**Fig. 1:**
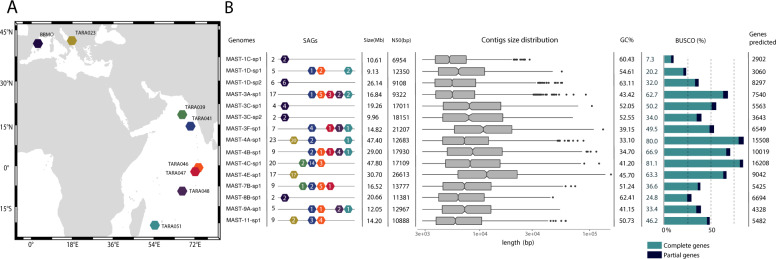
Genomic characteristics of 15 MAST species obtained by coassembling individual SAGs. **A** Location of marine sites where microbial communities were sampled. **B** Genome parameters of the 15 coassembled species: number of individual SAGs assembled and their distribution across sampling sites; assembled genome size; N50 assembly statistics and size distribution of contigs; GC content; genome completeness as the percentage of BUSCO complete (light blue) or fragmented (dark blue) gene models; and number of predicted genes.

Unicellular eukaryotic microorganisms were single cell sorted from planktonic assemblages in the Adriatic Sea and the Indian ocean during the Tara Oceans expedition, and in Spring 2018 from the BBMO (Fig. 1A). Based on their 18S rDNA signature, 140 cells from the unpigmented sort that affiliated to MAST lineages were selected for genome sequencing. Essential sampling and sequencing information regarding these SAGs is listed in Table S1. SAGs with similar tetranucleotide frequency and very high nucleotide similarity (fulfilling the criteria explained in M&M) were considered to be from the same species and combined into a coassembly, thus yielding improved genomes of 15 MAST species. The individual SAGs used in each coassembly often derived from different marine locations (Fig. 1B). Taking into account contigs ≥ 1 kb, we obtained genome sizes ranging from 9.13 to 47.80 Mb, each one with a characteristic GC content. Assembly quality assessments were carried out via the N50, the size distribution of contigs, and the genome completeness. The later, based on the percentage of conserved single-copy orthologous genes present in the final coassembly, averaged 46% across genomes, ranging from values as high as 80% in MAST-4A-sp1 and MAST-4C-sp1 to values as low as 7% in MAST-1C-sp1 (Fig. 1). Genomes with higher completeness also recovered more genes: 15,508 genes were predicted in MAST-4A-sp1, 16,260 in MAST-4C-sp1, and 2902 in MAST-1C-sp1. Thus, there was a clear correlation between genome size and both the genome completeness and the number of predicted genes. Overall, coassembled genomes provide reasonable gene completeness and represent a very promising resource to reveal the genes and the metabolic potential of uncultured MASTs. The 15 species for which we provide the new genomic data are widespread and relatively abundant in the global surface ocean (Fig. S2), thus representing useful targets to understand ecosystem functioning.

### Predicting the lifestyle of MAST species from genomics

**Fig. 2:**
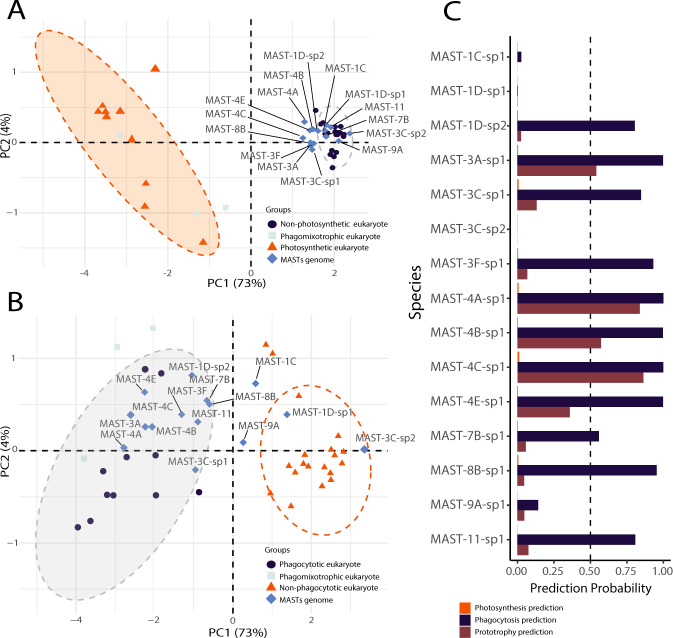
Lifestyle prediction of MAST species using a comparative genomics model [45]. **A** Plot of two first principal components (PC1 and PC2) placing genomes based on their genes associated to GO categories defining the photosynthetic lifestyle. **B** PCA plot placing genomes based on their genes associated to GO categories defining a phagocytotic lifestyle. **C** Prediction probabilities for MAST species to the three lifestyles. Dashed line ellipses in **A** and **B** illustrate 95% confidence assessments of the groupings based on photosynthetic and phagocytotic predictions.

We investigated the trophic lifestyle of the 15 MAST species using a recently published comparative genomics model [45]. Specifically, the training-based model interrogates the genomes of unknown species for the presence of genes predictive of phagotrophic, photosynthetic, or prototrophic lifestyles (Fig. 2). The model clearly predicted that none of the MAST species was photosynthetic: all of them were outside the photosynthetic PCA cluster, with 73% of the variation explained by the first principal component (Fig. 2A), and virtually zero prediction probabilities of being photosynthetic (Fig. 2C). Based on the set of genes defining phagotrophy, the majority of MAST species were placed with phagocytotic genomes (the first principal component explained 73% of the divergence) and within the 95% confidence ellipse in the PCA plot (Fig. 2B). The prediction probability for phagotrophy was above 80% in most cases, but it was very low in four of them, MAST-1C-sp1, MAST-1D-sp1, MAST-3C-sp2, and MAST-9A-sp1, precisely the ones that had the lowest number of predicted genes. At first sight, MAST species do not seem to perform prototrophy, being outside the prototrophic PCA cluster (Fig. S3). However, the species with most predicted genes (several MAST-4 and MAST-3A-sp1) display a moderate prediction probability to present this capacity (Fig. 2C).

Furthermore, while the previous analysis relied on preselected group of genes, we also performed a direct comparison of the 15 MAST species against a selection of other stramenopiles with known lifestyle (Fig. S1) using the number of genes in inferred OGs within each genome. The corresponding NMDS test revealed that the species grouped according to the defined trophic strategies: a tight photosynthetic cluster, an intermixed osmotrophic cluster, and a loose group including *Cafeteria burkhardae* and MAST species (Fig. S4). A PERMANOVA analysis showed that 22% of the variance in the plot (*p* < 0.001) was explained by the trophic mode, and this justified the use of the IndVal statistic to this dataset. Among the 28 OGs indicators of the phagocytosis trophic mode (Table 1), we identified many digestive enzymes (peptidases, glycosidases, lipases) and other genes related to cell growth and responses to the environment. A larger number of OGs characterized osmotrophs (Table S2) and phototrophs (Table S3), 133 and 744 OGs, respectively. In particular, phototrophs displayed many genes encoding for photosystem and other plastidic proteins.

**Table 1:** List of orthologous groups defining the phagocytotic lifestyle within the dataset of 30 stramenopile genomes.

Ortholog groups	IndVal	*p* value	InterPro	Description	GO term	General function
ORTHO03S000834	0.91	0.01	IPR011040	Sialidase	GO:0004553	Digestive enzyme
ORTHO03S000616	0.89	0.01	IPR004302	Cellulose/chitin-binding protein	–	Cell interactions
ORTHO03S000329	0.88	0.01	IPR004963	Pectinacetylesterase/NOTUM	GO:0016787	Digestive enzyme
ORTHO03S004730	0.87	0.01	IPR004981	Tryptophan 2,3-dioxygenase	GO:0019441	Digestive enzyme
ORTHO03S002955	0.83	0.01	IPR033396	Domain of unknown function DUF5107	–	Unknown function
ORTHO03S001168	0.83	0.01	IPR001577	Peptidase M8, leishmanolysin	GO:0008233	Digestive enzyme
ORTHO03S004520	0.83	0.01	IPR006201	Neurotransmitter-gated ion channel	GO:0034220	Membrane transport
ORTHO03S000334	0.82	0.03	IPR000884	Thrombospondin type 1 (TSP1) repeat	–	Cell interactions
ORTHO03S004517	0.79	0.01	IPR004911	Gamma interferon inducible lysosomal thiol reductase	–	Vacuolization
ORTHO03S004519	0.79	0.01	IPR016201	PSI domain	–	Cell adhesion
ORTHO03S005547	0.79	0.01	IPR002477	Peptidoglycan binding domain	–	Digestive enzyme
ORTHO03S002888	0.77	0.02	IPR011040	Sialidase	GO:0004553	Digestive enzyme
ORTHO03S003756	0.76	0.03	IPR021345	Protein of unknown function DUF2961	–	Unknown function
ORTHO03S004503	0.75	0.02	IPR012338	Beta-lactamase/transpeptidase like	GO:0005576	Digestive enzyme
ORTHO03S004518	0.75	0.01	IPR029787	Nucleotide cyclase	GO:0007165	Signal transduction
ORTHO03S004748	0.75	0.02	IPR036452	Ribonucleoside hydrolase	GO:0016614	Digestive enzyme
ORTHO03S005894	0.75	0.01	IPR008139	Saposin B type domain	–	Digestive enzyme
ORTHO03S004453	0.72	0.05	IPR017920	COMM domain	–	Regulation
ORTHO03S003676	0.72	0.03	IPR004007	Dihydroxyacetone kinase, subunit L	GO:0004371	Signal transduction
ORTHO03S005231	0.72	0.03	IPR004785	Ribose 5-phosphate isomerase B	GO:0005975	Sugar metabolism
ORTHO03S003865	0.72	0.04	IPR005524	Predicted permease DUF318	–	Membrane transport
ORTHO03S005235	0.71	0.02	IPR028730	Zinc finger FYVE domain-containing protein 26	GO:0061640	Cell division
ORTHO03S005554	0.71	0.03	IPR029723	Integral membrane protein GPR137	–	Transmembrane protein
ORTHO03S005577	0.71	0.01	IPR009613	Lipase maturation factor	–	Lipid metabolism
ORTHO03S005836	0.71	0.01	IPR001124	Lipid-binding serum glycoprotein	GO:0008289	Lipid metabolism
ORTHO03S005884	0.71	0.02	IPR002889	Carbohydrate-binding WSC	–	Cell interactions
ORTHO03S005895	0.71	0.02	IPR008139	Saposin B type domain	–	Digestive enzyme
ORTHO03S005965	0.71	0.01	IPR011124	Zinc finger, CW type	GO:0046872	Regulation

The OGs are first selected by the IndVal test (phagotrophs versus other genomes) and kept when their IPR identification was not found in the lists of OGs characterizing other lifestyles. The InterPro domain annotating each of the 28 OGs is shown, together with its description and a general function. When available the corresponding GO term is also provided.

**Fig. 3:**
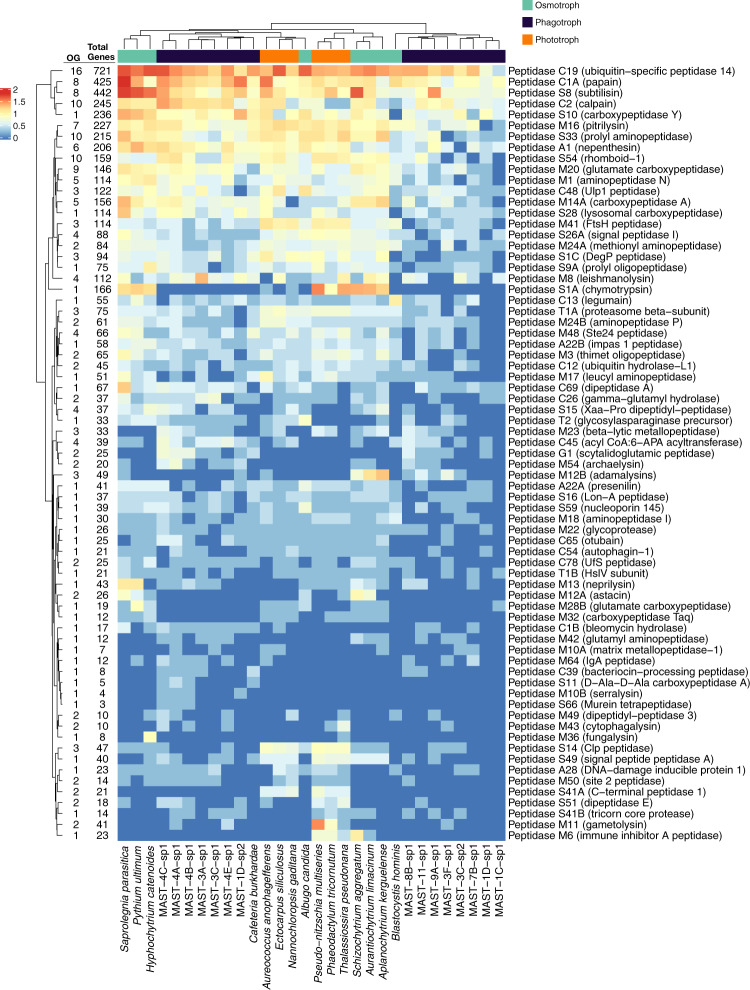
Distribution and abundance (log-transformed number of genes) of peptidase families in the 30 stramenopile genomes. Peptidase families follow the MEROPS classification (type enzyme in parenthesis) and may represent several OGs (number of OGs per family in the first column at the left of the heatmap) including many genes (overall number in the second column).

We focused on a given group of digestive enzymes, the peptidases, and explored how frequent they were among the complete set of stramenopile genomes. For this, we selected the 295 OGs that were functionally annotated as peptidases or proteases and studied their distribution in the 30 genomes, both at OGs level (Fig. S5) or after grouping OGs in 71 peptidase families (Fig. 3). These digestive enzymes were present in all species of phototrophs, osmotrophs, and phagotrophs in roughly similar gene copy numbers, around 250 genes on average per genome. Therefore, the number of peptidases genes could not be used as indicators of phagotrophic lifestyle. In the OGs heatmap (Fig. S5), the genomes clearly grouped by lifestyle (except *Blastocystis hominis* that appeared with phagotrophs) and some clusters accumulated OGs with IndVal scores, so seemed indicative of given lifestyles. However, in the heatmap constructed with peptidase families (Fig. 3), the grouping of genomes per lifestyle was less clear and a poor correlation of peptidase types and trophic mode was observed.

### Canonical proton pumps in their role of vacuole acidification

**Fig. 4:**
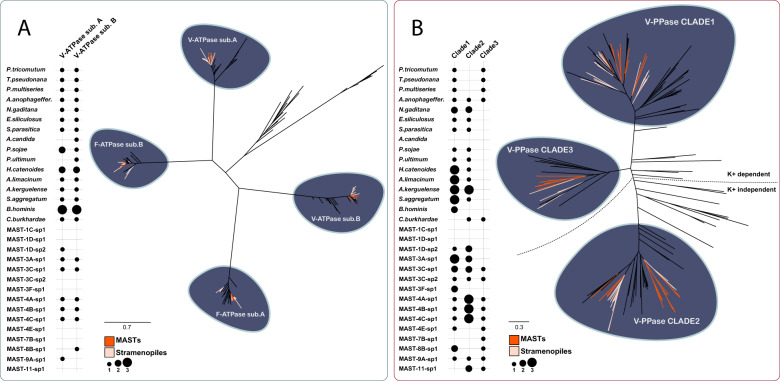
Phylogenetic representation of two distinct proton pumps across stramenopile genomes. The trees for V-ATPases (**A**) and V-PPases (**B**) are based on 185 and 184 protein sequences, respectively.

Vacuole acidification, a necessary step for the function of acidic digestive enzymes in mature phagosomes, is achieved by the action of the proton pump V-ATPase, and perhaps the V-PPase. We investigated the presence and the sequence homology of both genes in uncultured MASTs, other stramenopiles, and several other eukaryotes by phylogeny (Fig. 4). We first looked for the presence of the subunits A and B of the V-ATPase complex, which are homologous to the two subunits of the F-ATPase (Fig. S6). They were found in all complete genomes investigated here but were undetected in about half of the MAST species, most likely due to genome incompleteness. With respect to V-PPase, these were distributed in the three described clades: clade 1 homologous to the prokaryotic K^+^ dependent H^+^-PPases; clade 2 homologous to the prokaryotic K^+^ independent H^+^-PPases; and clade 3 related to the prokaryotic K^+^ dependent Na^+^ PPases (Fig. S7). Despite genome incompleteness, MASTs species show a remarkably high number of V-PPase genes, three on average, often within the three separate clades. Among them, MAST-4A-sp1, MAST-4B-sp1, and MAST-4C-sp1 contain a particular duplication of the clade 2 ancient to the divergence of the three species (Fig. S7). It is particularly interesting the presence of clade 3 V-PPase in MAST species, as this paralog is *often absent* in other eukaryotic genomes. In the stramenopile set studied here, oomycetes, labyrinthulomycetes, and the multicellular brown algae *Ectocarpus* appear to have lost clade 3, which is retained only in some diatoms and *C. burkhardae*. Finally, only two MAST species lacked V-PPase genes (MAST-1C-sp1 and MAST-1D-sp1), and this may likely be due to genome incompleteness.

### Rhodopsins and genes for retinal biosynthesis

**Fig. 5:**
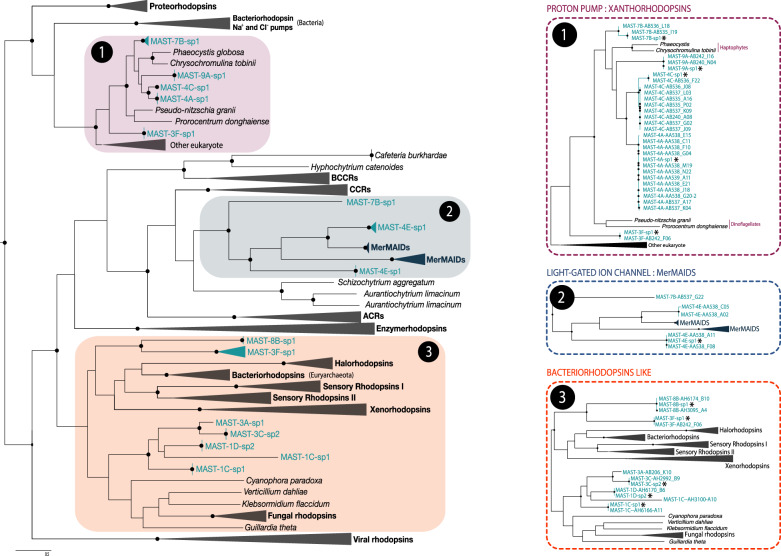
Phylogenetic tree of microbial type I rhodopsins based on 207 protein sequences, including the new MASTs, showing the recognized groups and their prevalent function. Black dots indicate bootstrap support > 80% over 1000 replicates. Stars highlight sequences recovered from coassemblies.

Rhodopsins are transmembrane proteins that together with a retinal pigment use light energy for proton translocation. Sequence similarity searches confirmed the presence of rhodopsin-like proteins in 11 of the 15 MAST genomes, typically found in multiple individual SAGs (Fig. S8). We carried out a phylogenetic analysis of the full range of microbial type I rhodopsins including also eukaryotic and viral sequences. The new MAST rhodopsin proteins were classified into distinct phylogenetic branches (Fig. 5). Some affiliated with the xanthorhodopsins type, which were already known in marine haptophytes, dinoflagellates, and diatoms. Xanthorhodopsins pump ions across cell membranes and contain carotenoid accessory pigments as a light-harvesting mechanism. With the exception of MAST-3F-sp1, in which only one of nine cells contained xanthorhodopsin (Fig. 5), this coding protein was found in several cells of MAST-4A-sp1, MAST-4C-sp1, MAST-7B-sp1, and MAST-9A-sp1. This strongly supports the idea that these rhodopsins truly belong to MAST species and are not a product of contamination.

A second clade revealed the presence in MAST species of the recently identified MerMAIDs rhodopsins. These light gated ion channelrhodopsins seem specific of marine microbes and were present in MAST-4E-sp1 (in several cells and featuring two distinct copies), as well as in a MAST-7B-sp1 cell with moderate bootstrap support (82%). The amino acid sequences of MAST MerMAIDs aligned very well with the original reports and revealed a well-conserved structure (Fig. S9). Similar to other microbial rhodopsins, it features seven transmembrane helices and the lysine Schiff base in the seventh helix where the retinal chromophore typically attaches (Fig. S9). The sequence from MAST-7B-G22 lacks part of the protein but still shows the retinal-binding lysine. The remaining MAST rhodopsins were included in a large bacteriorhodopsin-like clade. Those from MAST-8B-sp1 and MAST-3F-sp1 were closer to halorhodopsins (chloride pumps) and sensory rhodopsins generally limited to halophilic archaea, as well as to xenorhodopsins (inward H^+^-directed proton pumps). Those from MAST-1C-sp1, MAST-1D-sp2, MAST-3A-sp1, and MAST-3C-sp2 were closer to a large clade including fungal and bacterial rhodopsins. Our phylogenetic tree also shows that some species, i.e., MAST-3F-sp1 and MAST-7B-sp1, encode microbial rhodopsins from different clades, having putatively different functions. Overall, our data demonstrate that most of the MAST species studied here contain rhodopsins and reveal an important heterogeneity of this gene.

**Fig. 6:**
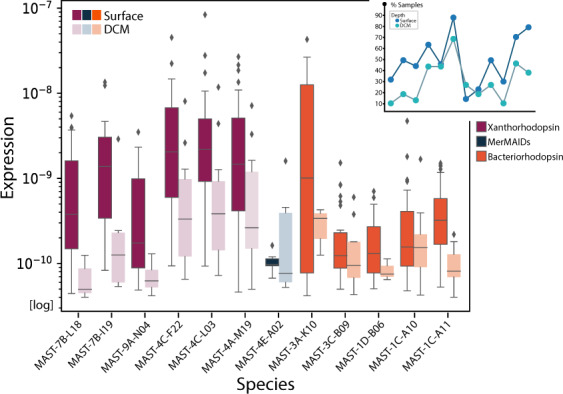
Expression level of MASTs rhodopsins in Tara Ocean metatranscriptomes (metaTs). The bar plot shows the gene expression in surface samples (57 metaTs) and DCM samples (36 metaTs) of each key rhodopsin gene based on its highly similar unigene of the MATOU dataset. The top upper-right inset indicates the percentage of samples in each water layer where each expressed transcript has been identified.

To broaden this statement, we looked at the expression level of the rhodopsin genes within the MATOU. Out of the 17 rhodopsin genes identified in the different MASTs (Fig. 5), 12 of them corresponded to a MATOU unigene (>98% similarity in an alignment >600 bp). We investigated the expression level of these unigenes in surface and deep chlorophyll maximum (DCM) metatranscriptomes from the 0.8–5 µm size fraction, the fraction where MAST cells are found (93 metaTs). This revealed that MAST rhodopsins from the three types were widely expressed in the epipelagic ocean (Fig. 6). The expression level of xanthorhodopsins was clearly larger at the surface than at the DCM. For the other two types, this depth difference was less obvious, although some bacteriorhodopsins exhibited more expression at surface than at DCM. Rhodopsin genes for which we could not demonstrate their expression in the ocean belonged to the three types. Intriguingly, the two genes of MAST-3F-sp1, a xanthoropdopsin and a bacteriorhodopsin, were not expressed.

**Fig. 7:**
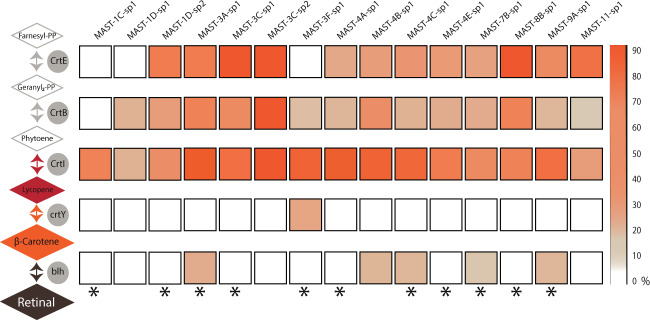
Presence of enzymes needed for retinal biosynthesis in MAST genomes: GGPP synthase (crtE), phytoene synthase (crtB), phytoene dehydrogenase (crtI), lycopene cyclase (crtY), and β-carotene 15,15′-dioxygenase (blh). The heatmap represents the proportion of individual SAGs within each species having the corresponding gene. Stars indicate species containing rhodopsins.

In addition to rhodopsins, we searched for the genes encoding the retinal biosynthetic pathway (Fig. 7 and Fig. S8). This pathway starts with the enzyme GGPP synthase (crtE), the last enzyme involved in isoprenoid biosynthesis, which produces geranyl_2_-PP. The next step involves the synthesis of phytoene from two geranyl_2_-PP, carried out by phytoene synthase (crtB), followed by a sequential desaturation and isomerization via phytoene desaturase (crtI) to synthetize lycopene. The enzymes crtE, crtB, and crtI are present in most of the studied MAST species and in many of the individual SAGs (Fig. S8). Synthesis of β-carotene is then catalyzed by the lycopene cyclase (crtY). The key and final step is the oxidative cleavage of β-carotene into retinal by the enzyme β-carotene 15,15′-dioxygenase (blh). This crucial step was detected in only a few MASTs, and the previous step partially found in a single one, which suggests that this pathway is not functional in MASTs. The gene retinal pigment epithelium-specific 65 kDa protein (RPE65), which encodes a protein for the regeneration of the 11-cis-retinal chromophore of rhodopsin in vertebrates, has been detected (Fig. S8).

## Discussion

### Obtaining reliable genomes of uncultured organisms by SCGs

In marine ecosystems, unicellular planktonic microbes typically have distinct trophic strategies placed in a trophic continuum mostly defined by energy transfer, from pure photosynthesis to prey uptake heterotrophy [57]. An important component of the marine plankton, the picoeukaryotes, is widespread, widely diverse, and includes multiple metabolic types [58, 59]. To date, the vast majority of heterotrophic picoeukaryotes cannot be cultured by traditional techniques, and this prevents the understanding of their functional traits, as both ecophysiological and genomic studies are not possible. SCG has proved to be reliable to recover genomic data from uncultured picoeukaryotes [16, 26, 60], to elucidate viral infections [61, 62] or phagotrophic interactions [63], and to highlight new evolutionary insights within animal multicellularity [64]. Here, we used SCG to obtain genome sequences and infer metabolic capacities of previously inaccessible MASTs. The new genomes of 15 MAST species, obtained by a coassembly strategy [16], showed a completeness often above 50%, higher to what is generally observed using single cells [65]. From these, we recovered a large number of predicted proteins per genome, the number of which generally correlates with genome size and completeness. While this represents a valuable culture-independent genomic resource, we cannot ignore the technical limitations of SCG. The necessary step of whole-genome amplification by MDA is well known to produce a patchy recovery of the original genome, which leads to fragmented and incomplete sequenced genomes that may affect subsequent analysis [14]. This can be partially alleviated (but not completely) by coassembling multiple cells. Thus, a gene not detected could be because it was absent in the genome or because it was lost during SAG generation and assembly. Nonetheless, we successfully provide genomic data from 15 uncharted branches of the stramenopile radiation, enabling us to access metabolic features and new physiological capabilities of MAST species.

### Predicting a general lifestyle for uncultured MASTs by comparative genomics

The placement of the MASTs at the base of the stramenopiles [7, 8], a phylogenetic region with a large diversity in life strategies including phagotrophy, osmotrophy, and parasitism, implies that the trophic roles of MAST species are not necessarily known. Here, we investigated the putative lifestyle of a phylogenetically varied set of MAST species using a recently published model based on comparative genomics [45]. The model showed evidence that MASTs do not have the proteins necessary for photosynthesis. Moreover, the genomic data strongly suggested that most of the MAST species have the faculty to perform phagocytosis. MAST-3C-sp2 and MAST-1D-sp1 clustered with photosynthetic eukaryotes when the model was trained with the proteins representative of phagocytosis, but this was probably due to the poor genome completeness of both species. In addition, the model seems unable to differentiate between phagocytotic and osmotrophic strategies, as osmotrophic species in the original publication (i.e., oomycetes, see Fig. S1 in [45]) as well as Hypochytrium and labyrinthulomycetes analyzed here (data not shown) were predicted to be phagocytotic. The grouping of osmotrophic genomes excluding MASTs in NMDS plots with complete gene data suggests that MAST species are phagotrophs and not osmotrophs. While the essential genes for photoautotrophy have been well documented either by comparative genomics or experimentally [66, 67], the identification of core proteins for phagocytosis is much less evident. Comparative proteomics have suggested a set of about 2000 proteins associated to the phagosomes [68]. However, the core genes associated to phagocytosis are still difficult to define [45] especially because these genes are used across multiple cellular functions. The assignment of a prototrophic lifestyle was also part of the model predictions, but we did not detect a high capacity to synthesize de novo low molecular-weight essential compounds in any MAST species, which might further support their dependency on phagocytosis.

### Challenges in the quest for exclusive phagotrophic genetic tool kits: peptidases, as example

As comparative genomics suggested that the MAST species investigated here were phagotrophs, we focused on genes putatively participating in the phagocytosis process. A previous study suggested distinctive functional capacities among heterotrophic picoeukaryotes, including some MASTs, related with glycoside hydrolases [26], here we emphasized the role of peptidases. As anticipated, peptidases appeared in every stramenopile genome tested. However, what was not expected is that both the number of peptidases per genome or the types of peptidases did not differ among trophic styles. The weak clustering of species by trophic strategy based on OGs (Fig. S5) could be due to the fact that species that share trophic role tend to be closer phylogenetically. Thus, the same peptidase family could form different OGs depending on the trophic mode. Correcting this effect by grouping OGs from the same peptidase family, we lose any pattern relating peptidases and trophic styles (Fig. 3). Thus, the amount and types of peptidases were similar in phagotrophic, phototrophic, and osmotrophic species. This is in agreement with the fact that all eukaryotic species contain lysosome-related organelles used in autophagic process that promote the turnover and degradation of their own proteins. Therefore, it is unlikely to find distinct types of peptidases exclusively associated to phagotrophy.

### High presence of V-PPases in MAST genomes

Extending our research toward the vacuole acidification, we focused on two widely known proton pumps: V-ATPases and V-PPases. V-ATPases are considered to be ubiquitous components of eukaryotic organisms, typically found in a single copy per genome, and are the canonical proton pumps for lysosome acidification [49, 69]. Accordingly, V-ATPases were found in all stramenopiles with complete genomes and in the majority of MAST species (Fig. 4A), with their absence likely being explained due to genome incompleteness. V-PPases were initially described as a proton pump that acidifies the lumen of vacuoles in land plants and microbial eukaryotes [70, 71]. Their role has been expanded to the acidification of the lumen of acidocalcisomes [20], an organelle that accumulates polyphosphate, calcium, and other cationic metals in green and red algae [20, 72] as well as in trypanosomatid and apicomplexan parasites [73]. A recent analysis on the evolution of V-PPases showed that they are absent in opisthokonts and amoebozoans [20], the eukaryotic supergroups in which most of our understanding of phagotrophy comes from [74]. In contrast, they are highly represented in MASTs species. The presence and, in some cases, concrete expansions of V-PPases in MASTs suggest an important role of this protein in modulating their cellular functions. In addition, clade 3 V-PPase seems to be particularly enriched in MASTs as compared to other stramenopiles with different trophic modes. It has been recently shown that *Cafeteria burkhardae* upregulates a clade 3 V-PPase when growing exponentially by bacterivory as compared to the stationary phase [75]. This suggests that these V-PPases, particularly from clade 3, may exert a key role in the vacuole acidification toward digestion in early-branching phagotrophic stramenopile clades.

### Extensive presence of rhodopsin genes in MAST genomes

Microbial rhodopsins are a diverse group of photoactive proteins capable of solar energy usage independent of plastid photosystems. They act as light-driven ion pumps or light sensors [76]. Homologs of these seven-helix transmembrane proteins have been reported in many prokaryotic taxa as well as in various eukaryotes, including marine species of diatoms, dinoflagellates [15, 77], haptophytes, cryptophytes [78], and MAST-4 [27]. Phylogenetic clades with putatively distinct functions have been identified [79]. Thus, homologs of the proton-pumping proteorhodopsins, initially found in marine bacteria [80], such as bacteriorhodopsins, halorhodopsins, sensory rhodopsins, and xanthorhodopsins [81], have been identified in archaea, bacteria, protists, and viruses [82]. Other types of microbial rhodopsins include fungal rhodopsins [83] and, lately, the channelrhodopsins known for its use in optogenetics [84]. Here, we extend the finding of diverse rhodopsins within uncultured MASTs belonging to distant stramenopile clades.

By themselves, rhodopsins are not photoactive: it is only when coupled with the light-sensitive retinal chromophore that they can convert light into an electrical response. The chromophore binds covalently to the rhodopsin domain through a Schiff base linkage with a lysine in the middle of the seventh helix [85], and we observed this conserved position at the right place in the alignments of MAST rhodopsins. The pathway of retinal generation involves two critical steps: the biosynthesis of β-carotene from its precursor lycopene, and the cleavage β-carotene into retinal [86]. The early steps of carotenoid biosynthesis to lycopene were present in MAST species but the genes involved in the last two critical steps were poorly recovered. This suggests that MASTs rely on their diet as a constant supply of retinal as these compounds cannot be synthetized de novo. An alternative explanation would be that MASTs take advantage of the presence of the RPE65 gene, known to catalyze the formation of retinal in vertebrates by an alternative biosynthetic pathway [87, 88].

We identified rhodopsins in most MAST species. Rhodopsins were not found in species with very uncomplete genomes (MAST-1D-sp1 and MAST-C-sp1) and in two species with an acceptable completeness (MAST-4B-sp1 and MAST-11-sp1). Particularly intriguing was the absence of rhodopsin in MAST-4B-sp1, as this gene was present in the other three MAST-4 species; further work is needed to confirm this absence. Five MAST species contained xanthorhodopsins, a subtype of light-driven proton pumps derived from halophilic bacteria that contain an additional light-harvesting carotenoid antenna [81]. They formed a highly supported cluster together with genes of marine haptophytes and dinoflagellates [77]. Xanthorhodopsins were the highest expressed rhodopsins in the ocean, especially in surface waters, suggesting a light dependency. Two species (MAST-4E-sp1 and MAST-7B-sp1) contained MerMAIDs rhodopsins, a new type recently discovered by metagenomics [51]. The MerMAIDs are closely related to cation channelrhodopsins but conduct anions, which make them unique. This is the first report of MerMAIDs rhodopsins in nonphotosynthetic protists. Non-MerMaiD channelrhodopsins were found in other stramenopiles such as *Hyphochytrium catenoides* [89]*, Cafeteria burkhardae*, *Schizochytrium aggregatum*, and *Aurantiochytrium limacinum* (Fig. 5). Channelrhodopsins are involved in light-sensing functions such as phototaxis in green algae [90], or even modulate the colony conformation of a choanoflagellate [91]. Thus, these rhodopsins might present a different function than xanthorhodopsins and bacteriorhodopsins, whose activity as proton pumps might complement the role of V-ATPase and potentially V-PPase in acidifying digestive vacuoles [28]. The fact that we observed a high expression of the xanthorhodopsin gene in MAST-4A when growing by bacterivory strongly support this hypothesis [27], but this still needs an experimental validation. With the observed widespread presence and gene expression of rhodopsins and the conserved transmembrane lysine for retinal binding, we suspect that light may play a more important role for phagotrophic MASTs than we originally thought. It is also interesting to note that some species harbor more than one rhodopsin type, suggesting independent acquisitions and complementary roles. Thus, the physiological capabilities conferred by different rhodopsin types might contribute to the various functions of MASTs in marine ecosystems. Describing them is the first step to create hypothesis and better understand functional differences between MAST species and clades.

## Conclusion

Due to their inability to be cultured, the physiology and ecology of many MAST species is still little understood. By genome sequencing of single eukaryotic cells, we bypassed cultivation requirements and gained insights into these neglected microbial eukaryotes. Comparative genomic analyses indicated a phagocytotic capability of these uncultured lineages. Genes clearly involved in phagocytosis, such as proton pumps for vacuole acidification and peptidases for prey digestion, were not exclusive of phagotrophic species and were equally represented in phototrophic and osmotrophic species. However, the remarkable presence of V-PPases and rhodopsins suggests that these proton pumps might play a crucial role in MAST species. Besides acidifying food vacuoles, a parallel scenario could be that MAST species couple rhodopsins proton pumping with the production of PPi by V-PPases. This coupled pathway would confer them an alternative energy source, as occurs in glucose metabolism of the parasitic *Entamoeba histolytica* that uses PPi instead of ATP [92]. A better clue of the involvement of proton pumps, digestive enzymes, and rhodopsins in phagocytosis is needed and new evidences can be derived from gene expression studies with cultured species [75] or natural assemblages [27]. Finally, even though the physiological role of rhodopsins in MASTs still needs to be elucidated, their ample presence in the genomes, conserved functional structure, and widespread expression in the surface ocean suggest that light might play an unexpected role in phagotrophic MAST species, contributing to vacuole acidification, mediating phototaxis, or even providing alternative energy sources. This light usage is consistent with the fact that MAST species are restricted to the upper photic region of the oceans [93, 94]. Overall, our data reveal a high metabolic plasticity of the MAST species analyzed here, which might facilitate their existence in the oceans as very abundant bacterial grazers.

## Supplementary information

supplementary legends

Figure S1

Figure S2

Figure S3

Figure S4

Figure S5

Figure S6

Figure S7

Figure S8

Figure S9

Table S1

Table S2

Table S3

## Data Availability

Sequencing reads have been deposited at the GenBank Database under Project numbers PRJEB6603 for Tara SAGs and PRJEB41235 for BMMO SAGs. Additional data have been deposited in Figshare under the project number 10.6084/m9.figshare.c.5008046, including genome coassemblies, CDS predictions, alignments and phylogenetic trees, and used scripts. Individual SAGs, coassembled contigs, predicted genes, and proteins can also be explored through an in-house developed web repository (sag.icm.csic.es).
