# Supplementary material for: Comparative genomics reveals new functional insights in uncultured MAST species
Source: ISME J. 2021 Jan 15;15(6):1767–81. doi: 10.1038/s41396-020-00885-8 (PMC8163842; doi:10.1038/s41396-020-00885-8)
Supplement: Supplementary file 1 — supplementary legends [file 41396_2020_885_MOESM1_ESM.docx]

**Supplementary Information**

**Fig. S1.** Phylogenetic tree of the taxa used for comparative genomics analysis, including the 15 uncultured MAST species, using the 18S rDNA gene. The tree was generated with IQTREE using 1000 trees for topology and 1000 trees for bootstrapping. Five non-stramenopile taxa were used as outgroup. Eukaryotic species were assigned to a trophic lifestyle.

**Fig. S2.** Representation of the global distribution of MAST species at surface in the Malaspina dataset. Red dots represent the relative abundance (grey dots imply absence), and the histograms the abundance-spectra plots. Data from Logares et al. (2020) [94].

**Fig. S3.** Prototrophy prediction of MAST species using a comparative genomics model [45]. PCA plot placing genomes based on their genes associated to GO categories defining a prototrophic lifestyle.

**Fig. S4**. NMDS plot relating the 30 Stramenopiles genomes based on their Bray-Curtis dissimilarity calculated from the relative abundance of genes per genome within defined orthologous groups. The species are colored and grouped with a shadowed area according to their trophic lifestyle.

**Fig. S5.** Distribution and abundance (log-transformed number of genes) of OGs annotated as peptidases in the 30 stramenopile genomes. Taxa are grouped according to their trophic strategy (upper part of the graph), while some of the OG clusters also indicate a given trophic lifestyle, as marked by the accumulation of OGs with IndVal scores. Filtered IndVal indicate those OGs which IPR code was not found within the other IndVal sets.

**Fig. S6.** Phylogenetic tree of V-ATPases and the related F-ATPases genes constructed from recent bibliographical references (see Material and Methods). MASTs lineages are represented in orange. Values at nodes correspond to bootstraps > 80%.

**Fig. S7.** Phylogenetic tree of V-PPases genes constructed from recent bibliographical references (see Material and Methods). MASTs lineages are represented in orange. Values at nodes correspond to bootstraps > 80%.

**Fig. S8.** Presence of the genes needed for retinal biosynthesis in every individual MAST SAG. The presence of an enzyme for an alternative pathway (RPE65), as well as the presence of rhodopsin genes, is also indicated.

**Fig. S9.** Sequences alignment of MerMAIDs channelrhodopsins. Highly conserved amino acids are shown in orange (identical) and light blue (in more than 60% of the sequences). The α-helices 1-7 were determined based on a previous publication [51]. The lysine Schiff base for retinal attachment found in the 7th helix is identified in dark blue.

**Table S1.** Information related to the single cells used in this study: geographic provenance (Tara stations or the BBMO [Blanes Bay Microbial Observatory]); sampling depth (S: surface layer, D: Deep Chlorophyll Maximum layer); sequencing effort; Illumina platform and sequencing service used (GS: Genoscope, OR: Oregon Health & Science University, or CNAG: Centre Nacional d'Anàlisi Genòmica); its use for co-assemblies; reference for the genome.

**Table S2.** List of orthologous groups defining the osmotrophic lifestyle within the dataset of 30 stramenopile genomes. These OGs are first selected by the IndVal test (osmotrophs versus other genomes) and kept when their IPR identification was not found in the lists of OGs characterizing other lifestyles. The InterPro domain annotating each OG is shown.

**Table S3.** List of orthologous groups defining the photosynthetic lifestyle within the dataset of 30 stramenopile genomes. These OGs are first selected by the IndVal test (photosynthetic versus other genomes) and kept when their IPR identification was not found in the lists of OGs characterizing other lifestyles. The InterPro domain annotating each OG is shown.
