# Supplementary figures and images for: Comparative genomics reveals new functional insights in uncultured MAST species
Source: ISME J. 2021 Jan 15;15(6):1767–81. doi: 10.1038/s41396-020-00885-8 (PMC8163842; doi:10.1038/s41396-020-00885-8)

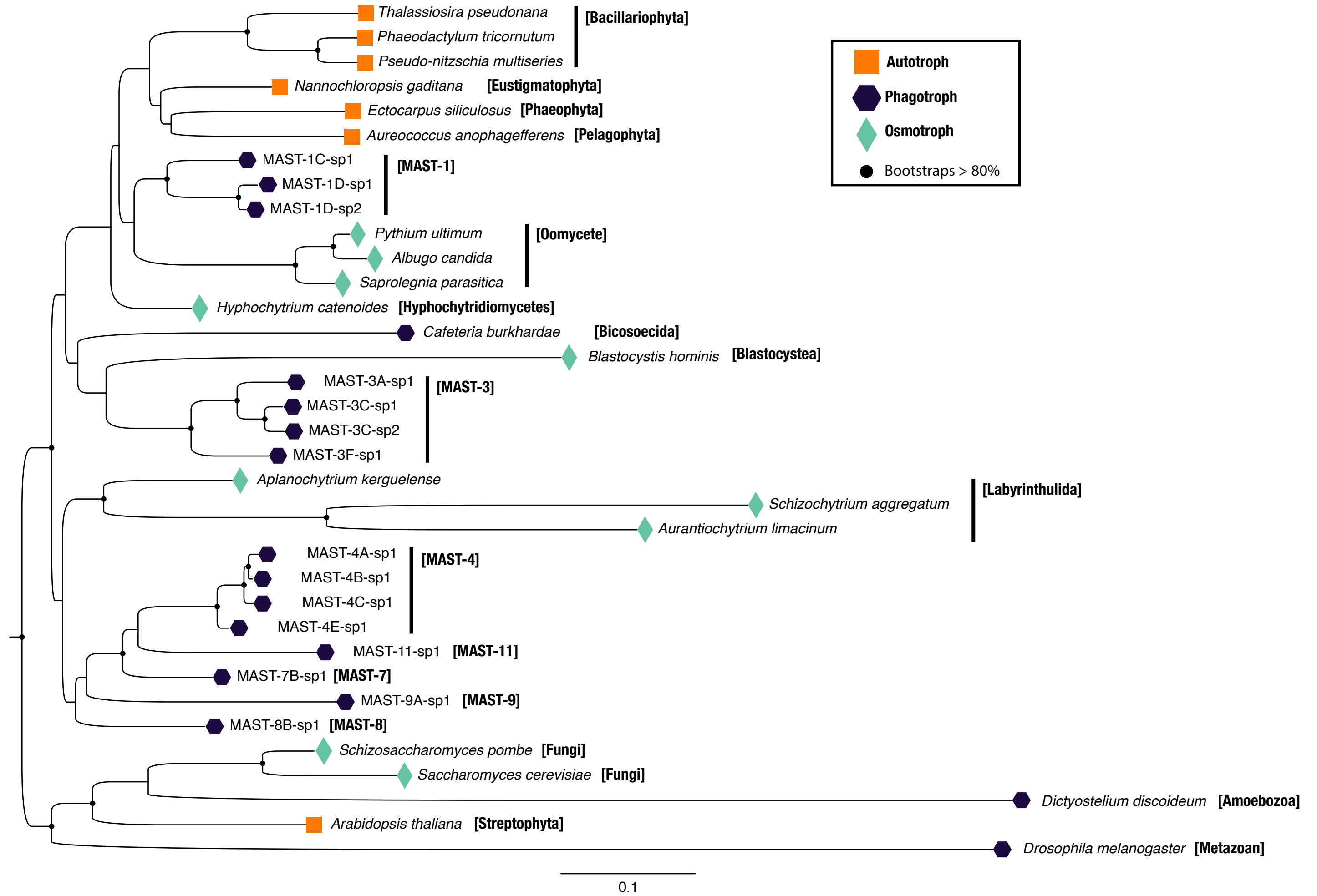

Supplement: Supplementary file 2 — Figure S1 [file 41396_2020_885_MOESM2_ESM.pdf]

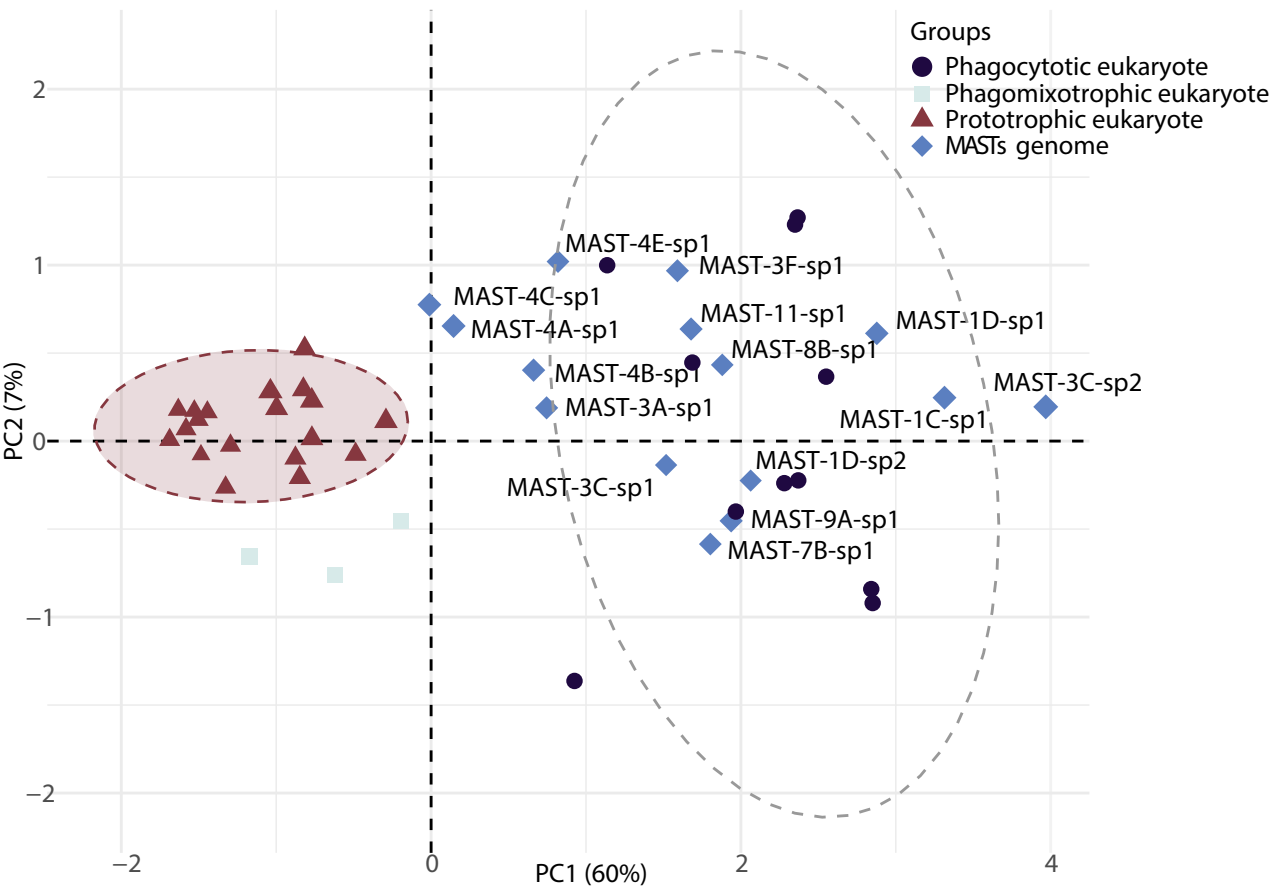

Supplement: Supplementary file 4 — Figure S3 [file 41396_2020_885_MOESM4_ESM.pdf]

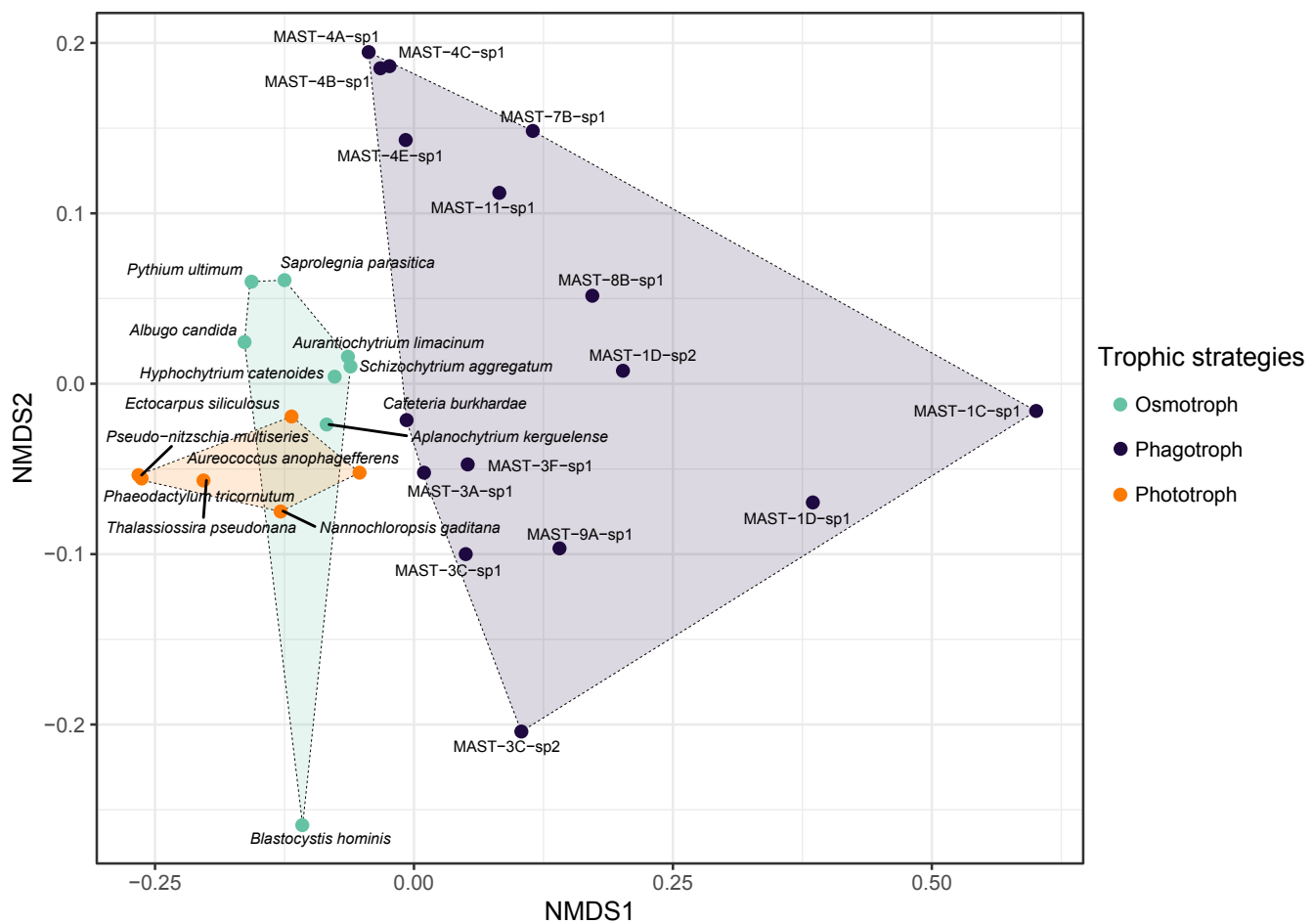

Supplement: Supplementary file 5 — Figure S4 [file 41396_2020_885_MOESM5_ESM.pdf]

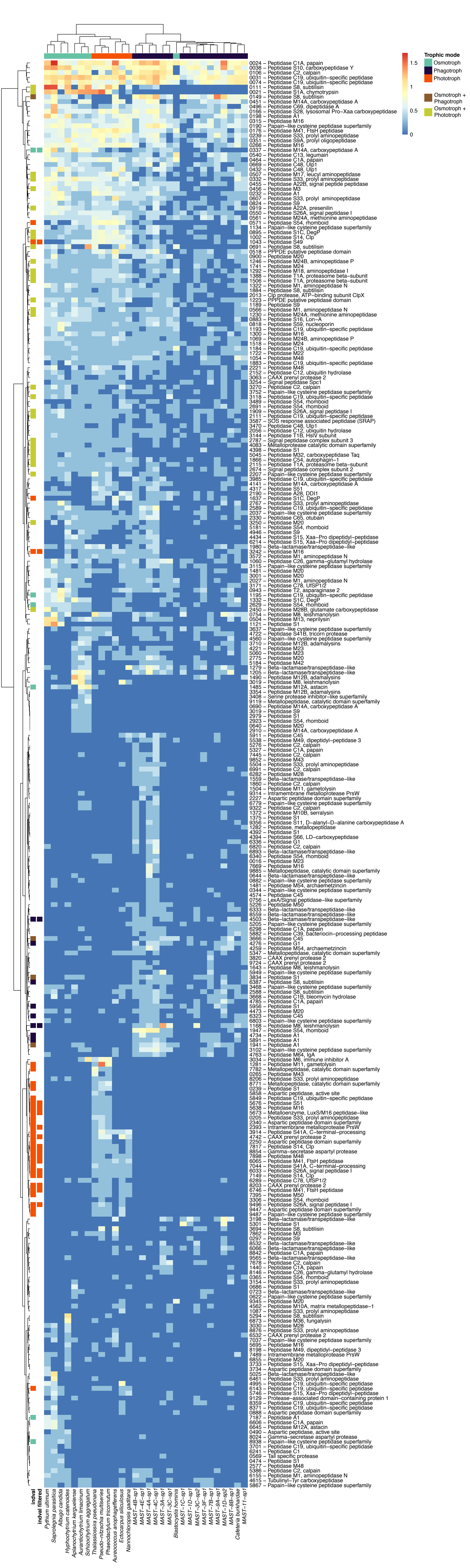

Supplement: Supplementary file 6 — Figure S5 [file 41396_2020_885_MOESM6_ESM.pdf]

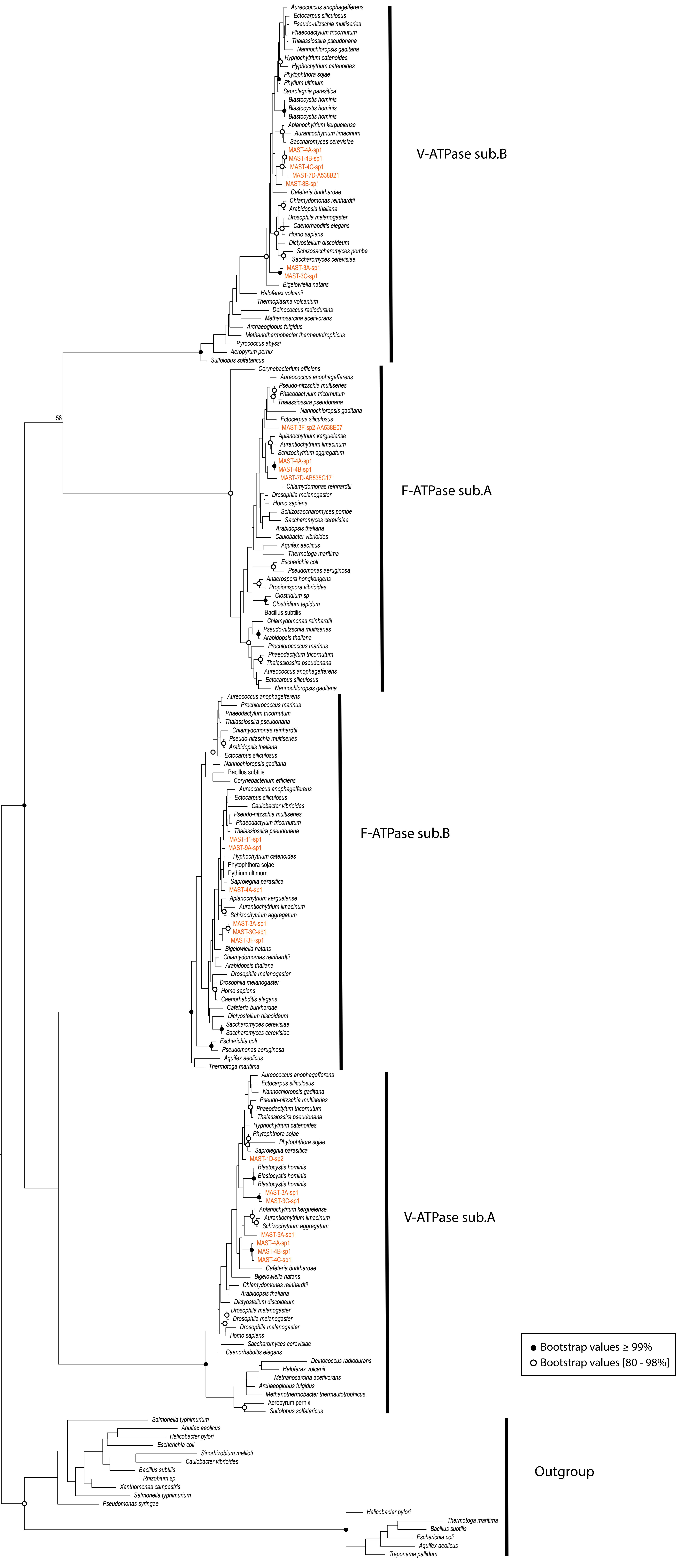

Supplement: Supplementary file 7 — Figure S6 [file 41396_2020_885_MOESM7_ESM.pdf]

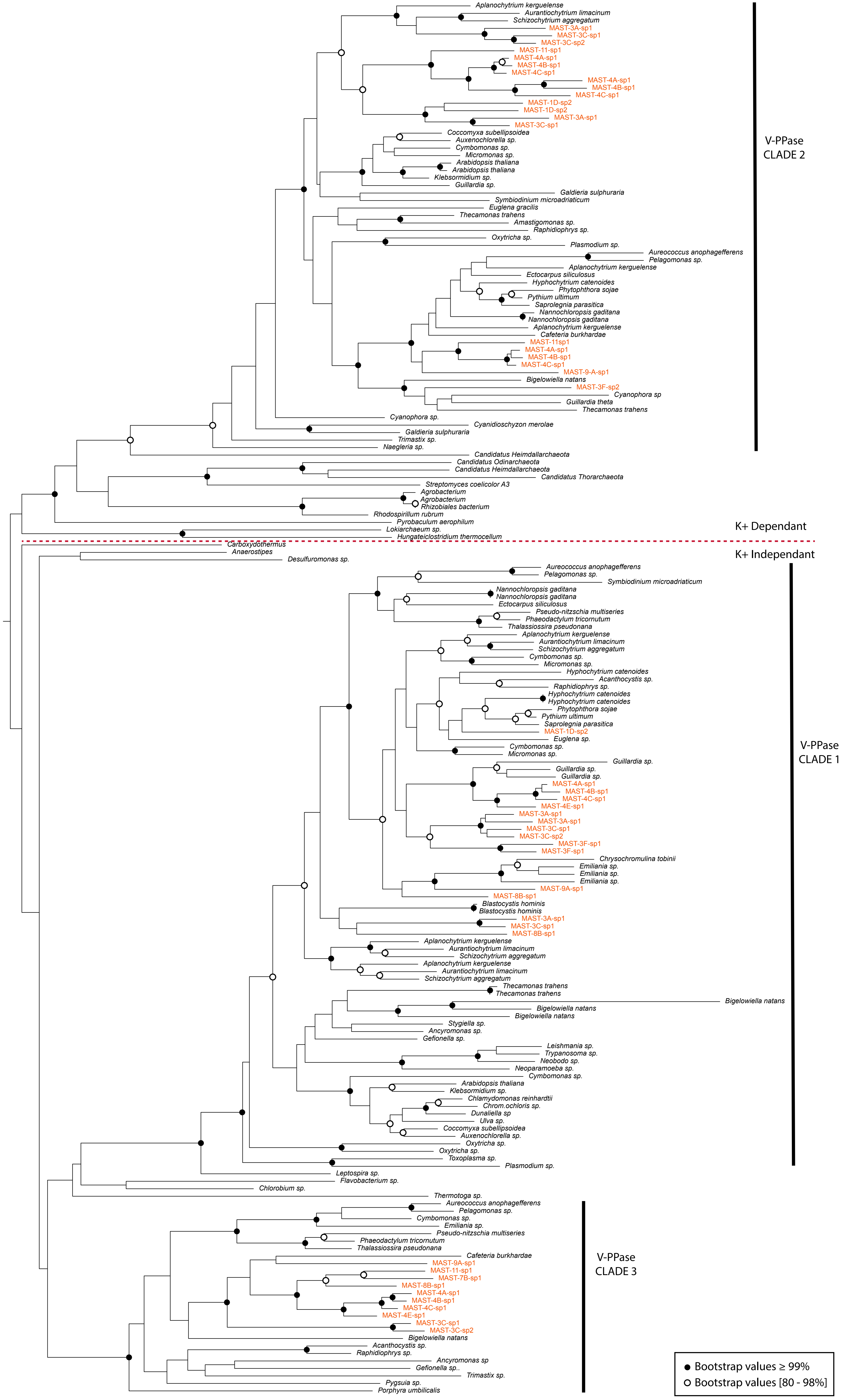

Supplement: Supplementary file 8 — Figure S7 [file 41396_2020_885_MOESM8_ESM.pdf]
