# Supplementary material for: Comparative genomics reveals new functional insights in uncultured MAST species
Source: ISME J. 2021 Jan 15;15(6):1767–81. doi: 10.1038/s41396-020-00885-8 (PMC8163842; doi:10.1038/s41396-020-00885-8)
Supplement: Supplementary file 3 — Figure S2 [file 41396_2020_885_MOESM3_ESM.pdf]

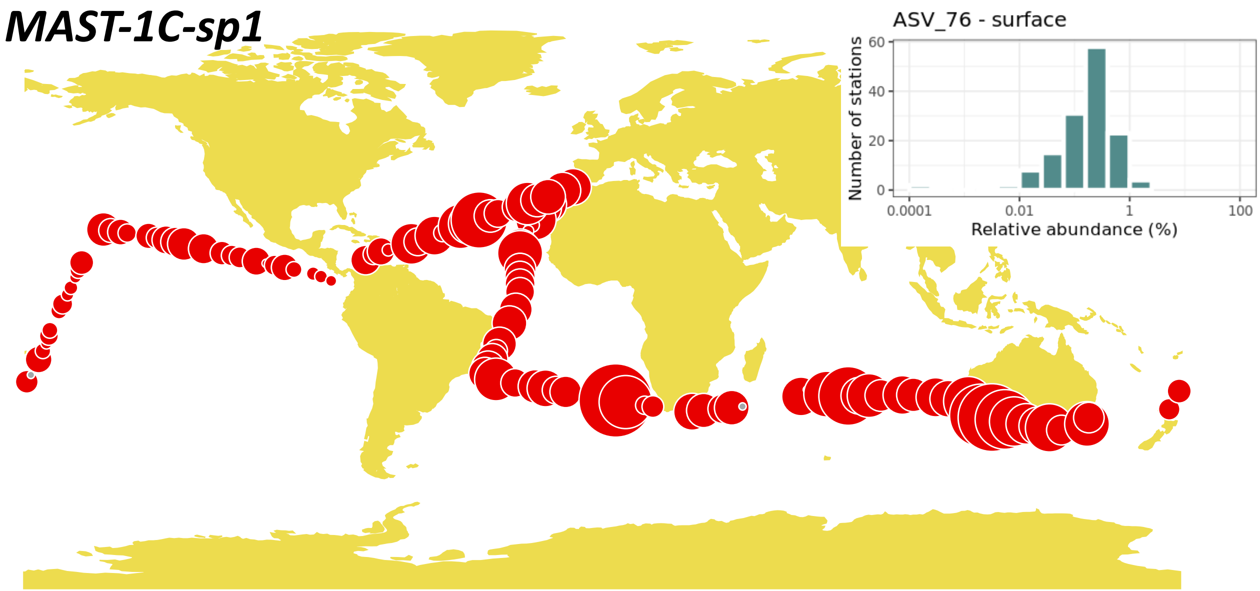
MAST-1C-sp1
ASV_76 - surface
Number of stations
60
40
20
0
0.0001
0.01
1
100
Relative abundance (%)

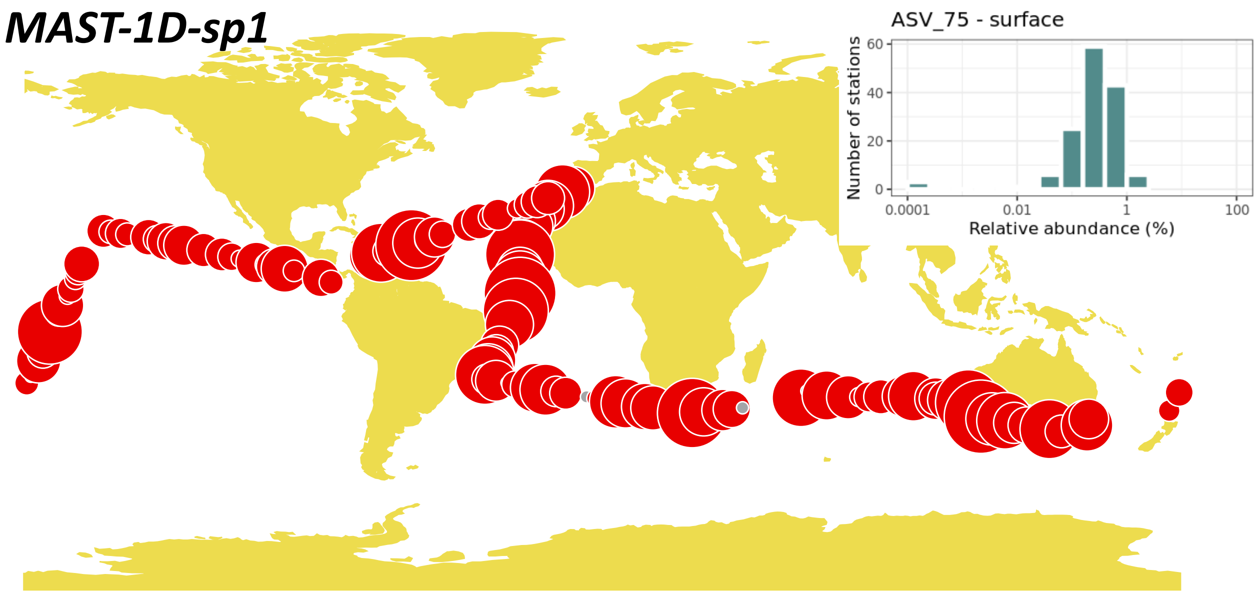
MAST-1D-sp1
ASV_75 - surface
Number of stations
60
40
20
0
0.0001
0.01
1
100
Relative abundance (%)

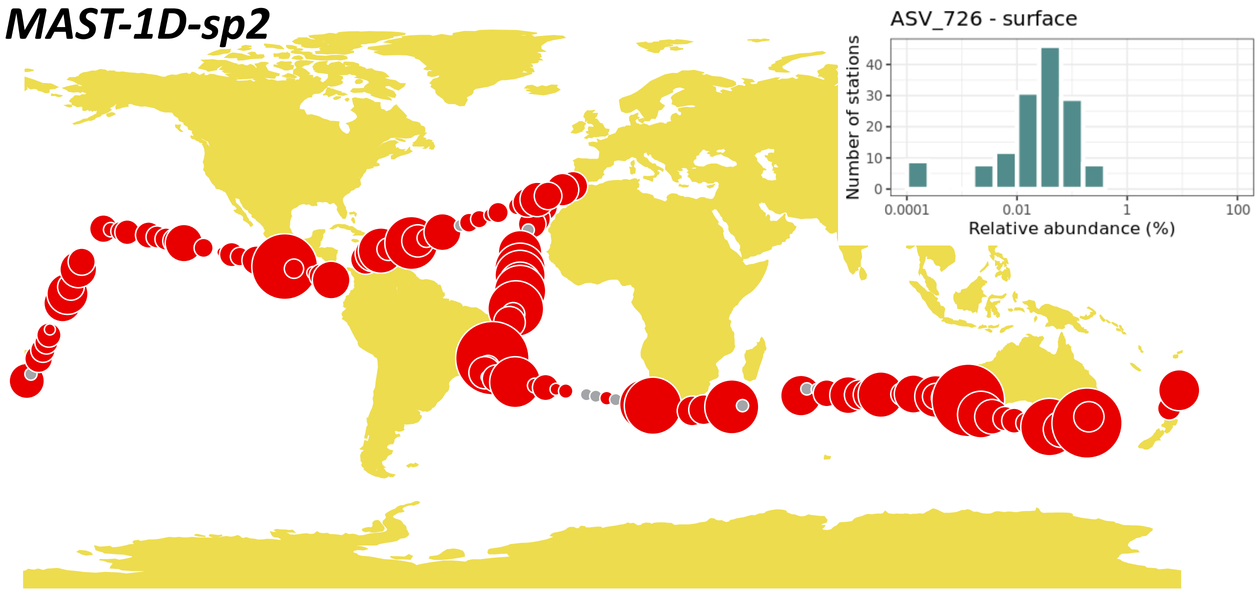
MAST-1D-sp2
ASV_726 - surface
Number of stations
40
30
20
10
0
0.0001
0.01
1
100
Relative abundance (%)

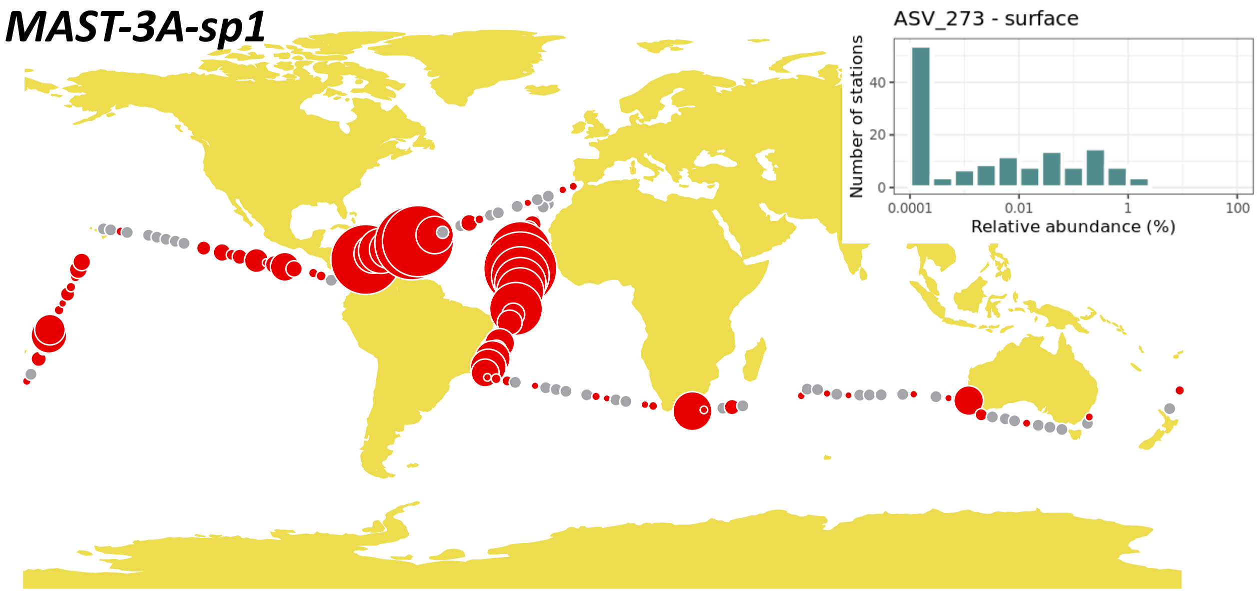
MAST-3A-sp1
ASV_273 - surface
Number of stations
0
20
40
0.0001
0.01
1
100
Relative abundance (%)

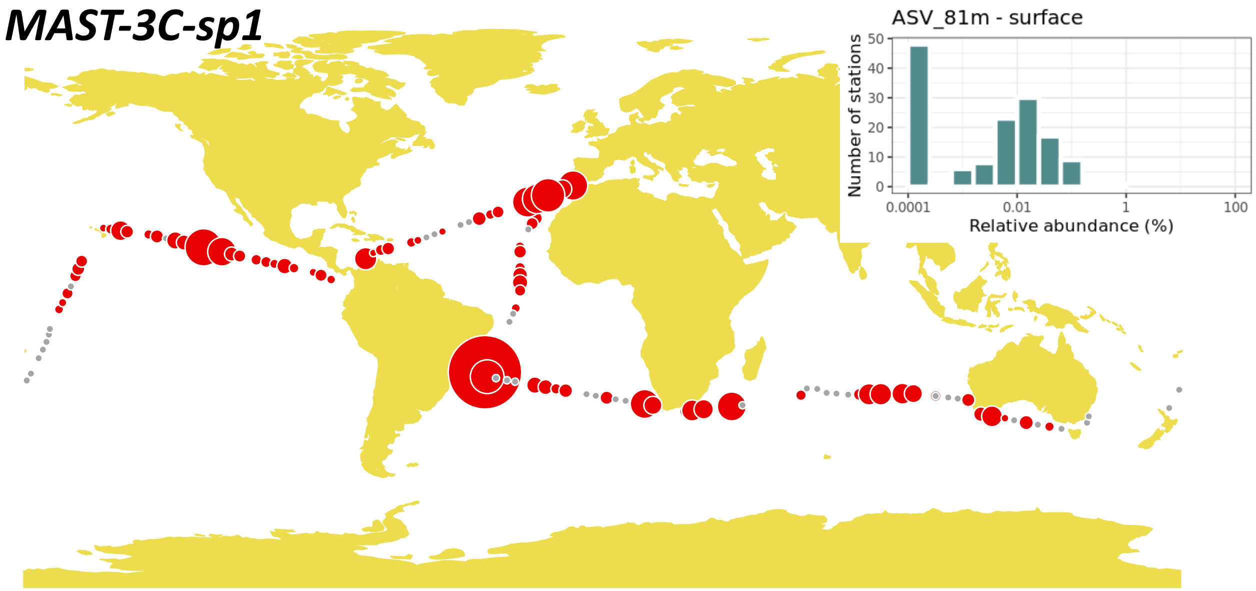
MAST-3C-sp1
ASV_81m - surface
Number of stations
0
10
20
30
40
50
0.0001
0.01
1
100
Relative abundance (%)

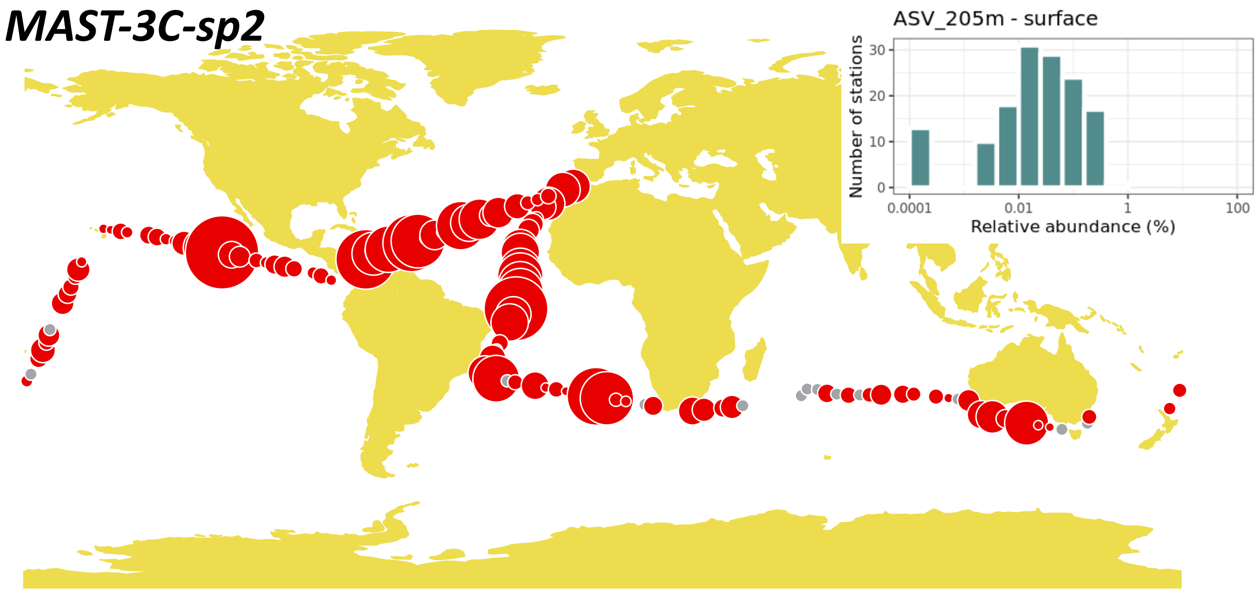
MAST-3C-sp2
ASV_205m - surface
Number of stations
0
10
20
30
0.0001
0.01
1
100
Relative abundance (%)

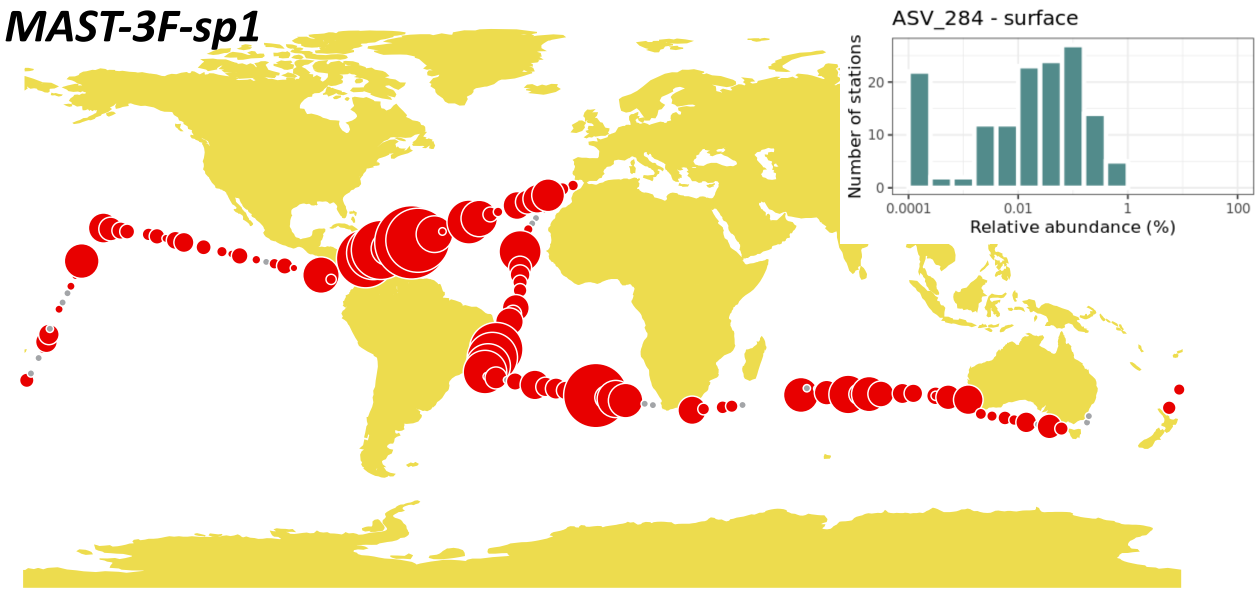
MAST-3F-sp1
ASV_284 - surface
Number of stations
0
10
20
0.0001
0.01
1
100
Relative abundance (%)

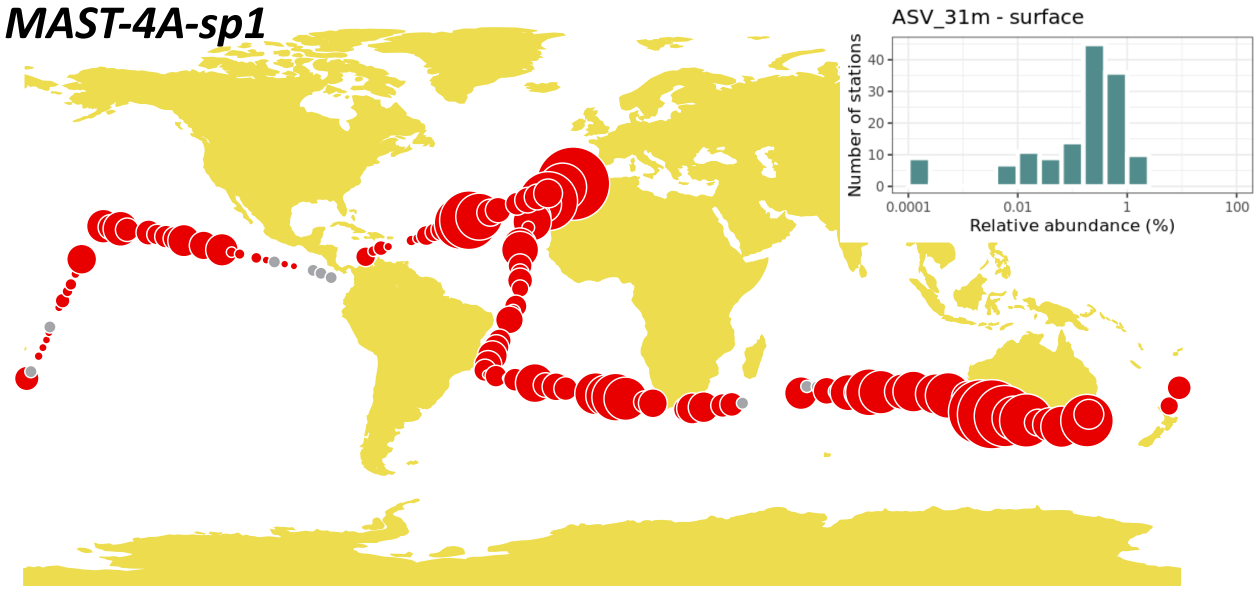
MAST-4A-sp1
ASV_31m - surface
Number of stations
0
10
20
30
40
0.0001
0.01
1
100
Relative abundance (%)

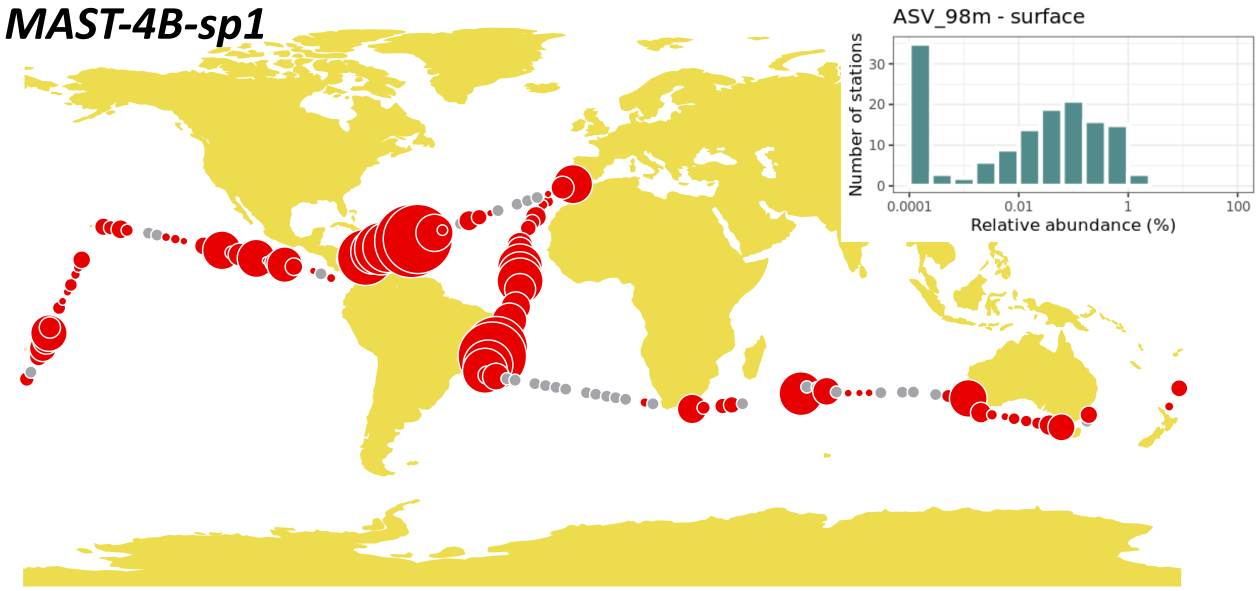
MAST-4B-sp1
ASV_98m - surface
Number of stations
0
10
20
30
0.0001
0.01
1
100
Relative abundance (%)

MAST-4C-sp1

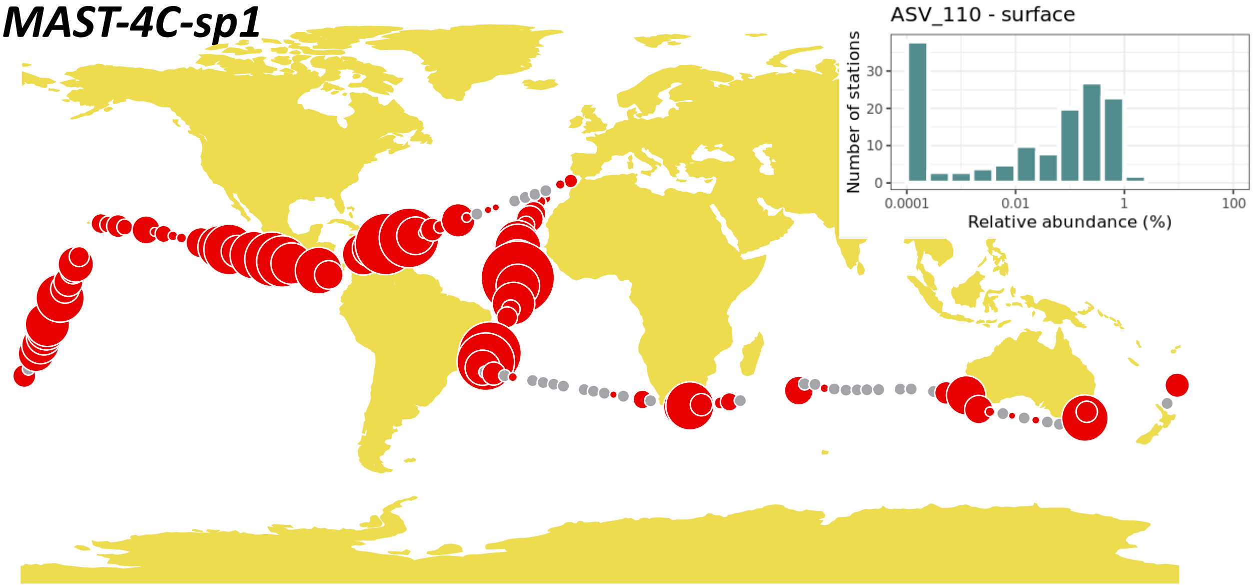

MAST-4E-sp1

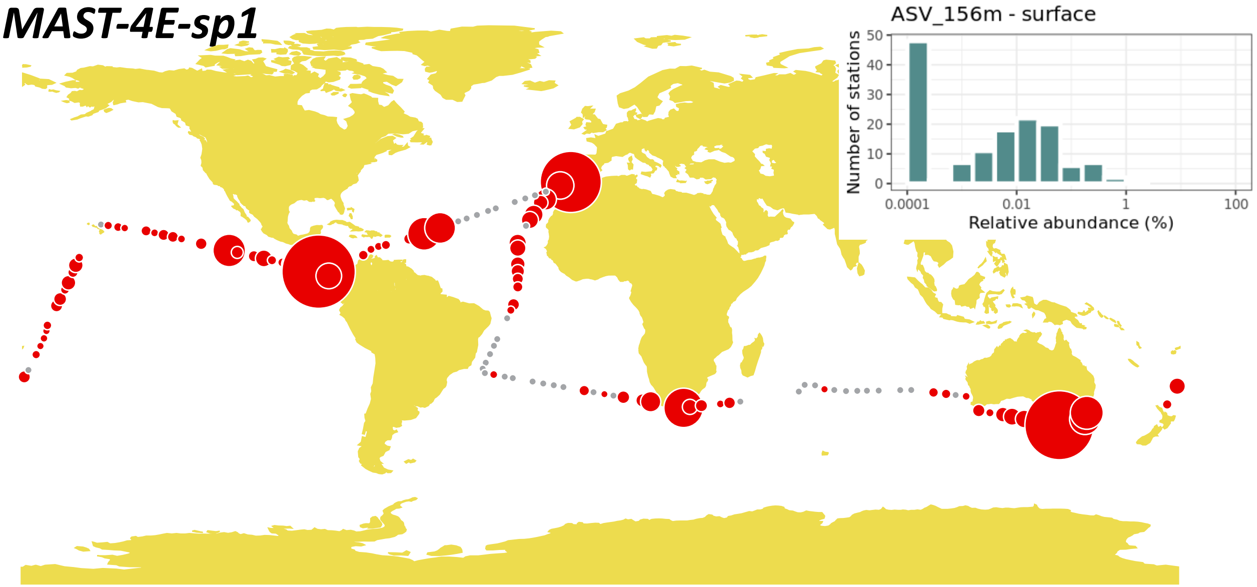

MAST-7B-sp1

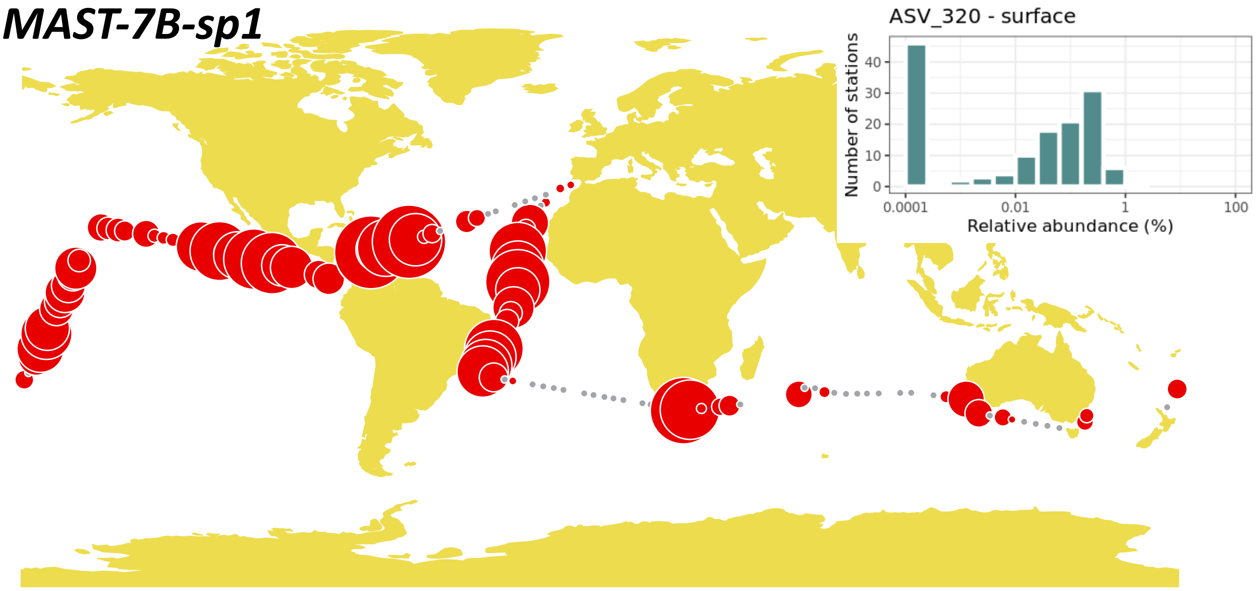

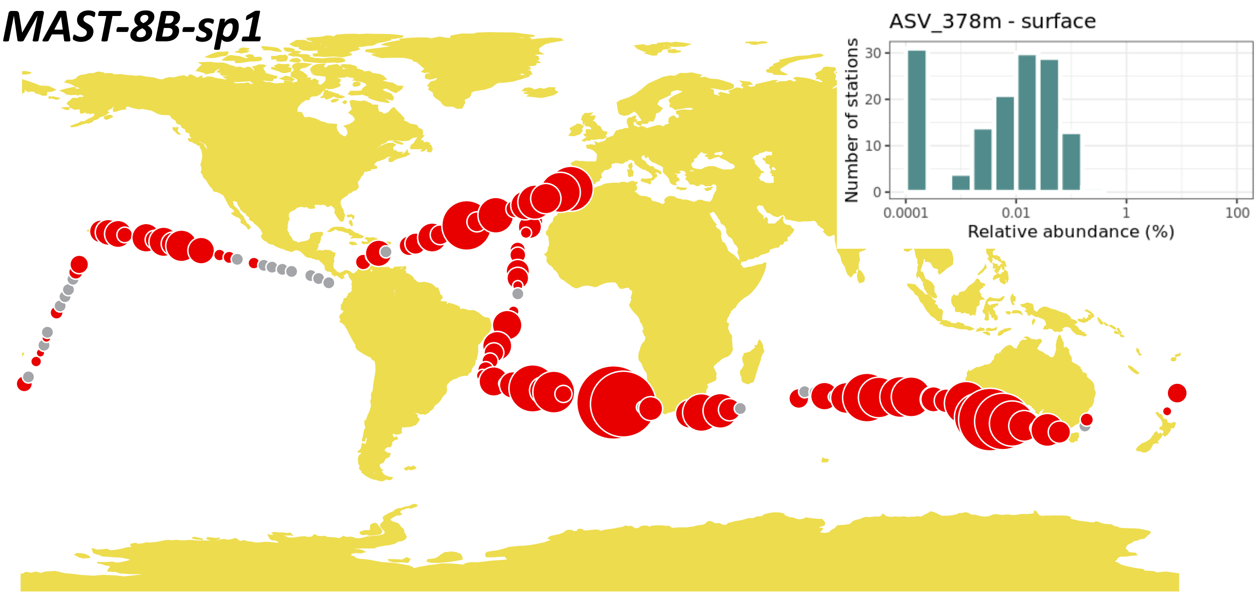
MAST-8B-sp1
ASV_378m - surface
Number of stations
0
10
20
30
0.0001
0.01
1
100
Relative abundance (%)

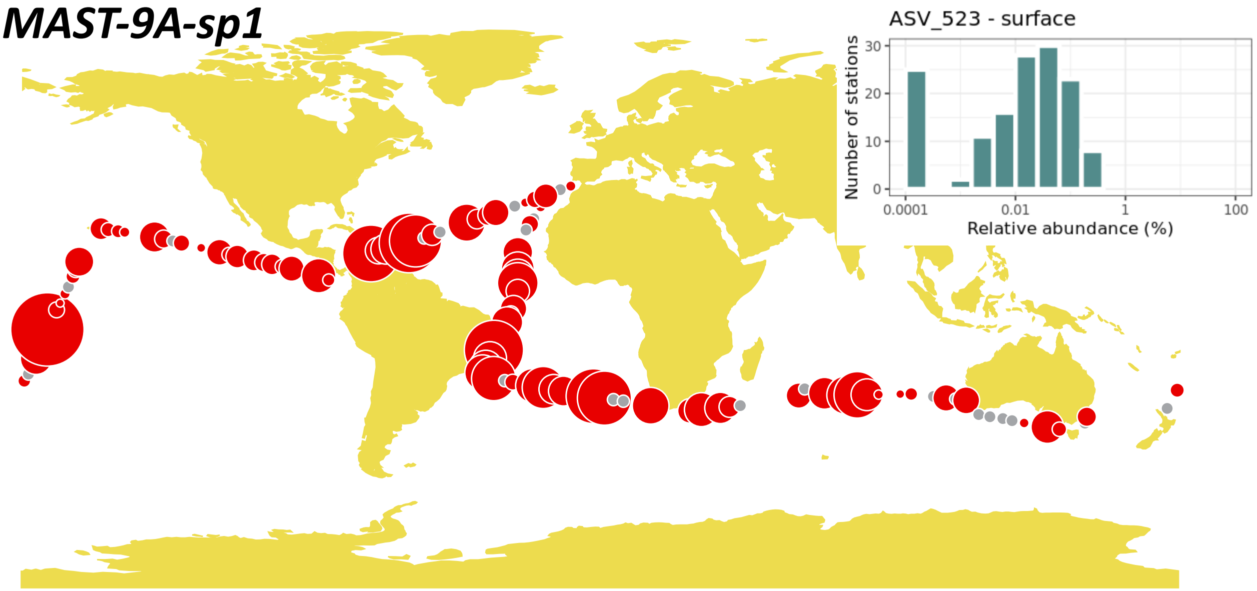
MAST-9A-sp1
ASV_523 - surface
Number of stations
0
10
20
30
0.0001
0.01
1
100
Relative abundance (%)

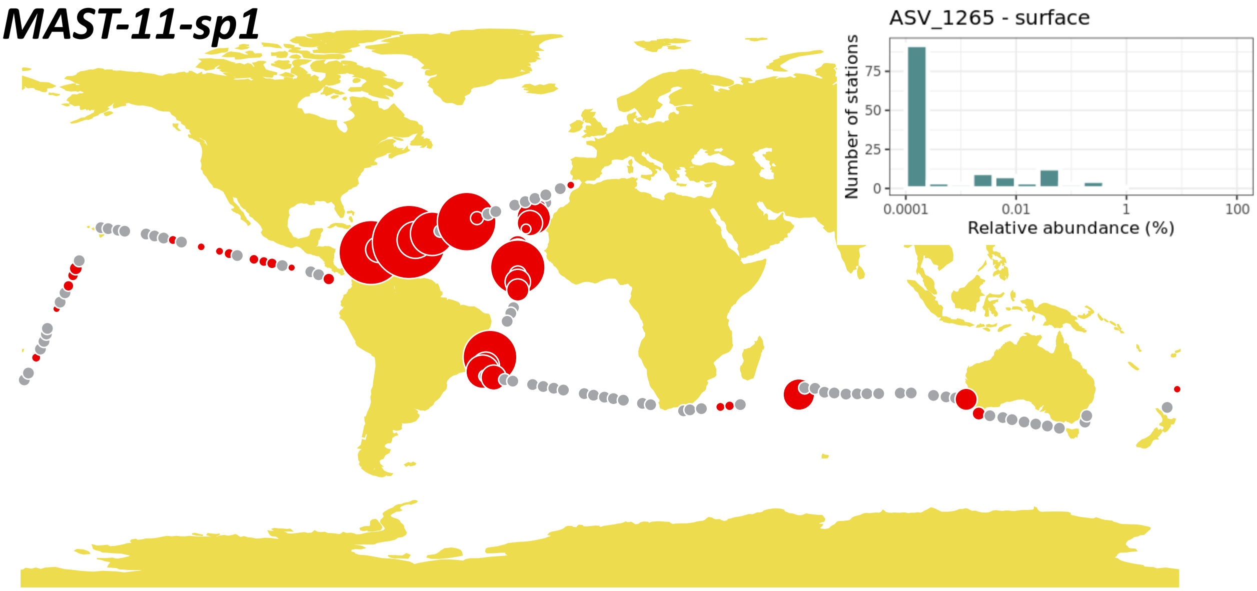
MAST-11-sp1
ASV_1265 - surface
Number of stations
0
25
50
75
0.0001
0.01
1
100
Relative abundance (%)
