# Supplementary material for: Comparative genomics reveals new functional insights in uncultured MAST species
Source: ISME J. 2021 Jan 15;15(6):1767–81. doi: 10.1038/s41396-020-00885-8 (PMC8163842; doi:10.1038/s41396-020-00885-8)
Supplement: Supplementary file 10 — Figure S9 [file 41396_2020_885_MOESM10_ESM.pdf]

```
MerMAID 1    IYATFQLFAVLITAATLVFYVSQLRNGNKG---DWEPIYVSGVEFIAYLMALVLPDGYAFRSFQDDSGI---ITVPVFRYVSWLATCPIILKVL
MerMAID 2    LHVFAQILTTFISCANAMYYLYIYKCTKRGHLMKWEVAWVAIVETINYTIQIST--GSEQIELAGGA------KFPWMRYVGWQLTCPVLLTFI
MerMAID 3    IYATFQLFAVLITAATLVFYVSQLRNGNKG---DWEPIYVSGVEFIAYLMALVLPDGYAFRSFQDGAGI---ITVPVFRYVSWLATCPIILKVL
MerMAID 4    IYATFQLFAVLITAATLVFYVSQLRNGNKG---DWEPIYVSGVEFIAYLMALVLPDGYAFRSFQDGAGI---ITVPVFRYVSWLATCPIILKVL
MerMAID 5    IYATFQLFAVLITAATLVFYVSQLRNGNKG---DWEPIYVSGVEFIAYLMALVLPDGYAFRSFQDDAGI---ITVPVFRYVSWLATCPIILKVL
MerMAID 6    LHVTAQIFTAFVSAANALYYFYIWRFTKRGRLMQWEVAWVAWVETINYVVQIAT--GSPRVGLIHGG------KFPWMRYVGWQLTCPVLLAFI
MerMAID 7    LHVFAQIFTAFVSAANALYYFYIYRCTKRGHLMKWEVAWVASVETVNYVIQVAT--GSPRVELAHGG------MFPWMRYVGWQLTCPVLLAFI
MAST-4E-F08  LHTAVQFMVFIVSFMSFVFYGYQKWQGQSR--VDWEIIYVVGIEVVLYSLLMGLPEEYTNIN---GA-------IPILRYMSWLTTCPVLLSNV
MAST-4E-A11  LHTAVQFMVFIVSFMSFVFYGYQKWQGQSR--VDWEIIYVVGIEVVLYSLLMGLPEEYTNIN---GA-------IPILRYMSWLTTCPVLLSNV
MAST-4E-A02  VHSTFQLFAVIATGGTLIFYTLQIRSGKRG---GWEPVYVSMVEFLAYFISFLLPDEYSFLSL----GER---SVPVFRYVSWLTTCPIILKVL
MAST-4E-C05  -----------------------------------------MIEFLAYFISFLLPDEYSFLPL----GER---SVPVFRYVSWLTTCPIILKVL
MAST-7B-G22  -------------------------------------------------------------------------------------------MMT

MerMAID 1    VTVISHEDN-IDQDVILRLMLLITWIELLGFMGSMYVGM-LKVFCIALAIILCIYMYYIICTTWNKNHHESL--------LLSYPKERFELMAL
MerMAID 2    VTNMLEAP---TTRVVVQMLMLLQFIMLSGMTASLLQAMTLKVIFVLLASAGLICLYYSLYTKR---KNKQL--------PMANNKE-MHLLLY
MerMAID 3    VTVISHEDN-IDQDVILRLMLLITWIELLGFMGSMYVGM-LKICFIALAVILCIYMYYIICTTWNKNHHKSL--------LLSYPKERFELMAL
MerMAID 4    VTVISHEDN-IDQDVILRLMLLITWIELLGFMGSMYVGM-IKVFFIALAVILCIYMYYIICMTWNKNHHESL--------SLSYRKERFELMSL
MerMAID 5    VTVISHEDN-IDQDVILRLMLLITWIELLGFMGSMYVGM-IKVFFIALAVILCIYMYYIICMTWNKNHHESL--------SLSYRKERFELMSL
MerMAID 6    VLNMLEKP---STRLVIQLLMLLQFIILSGMSASLLADNGLKVLFVIIASLGLCIMYSVLYTNR---KNKHV--------PSSENKE-LHLLMY
MerMAID 7    VVNMLESP---STRLVIQMLMLLQFIILSGMTASLVADMSMKILFVVIAALSLCILYYQLYTHR---KYKDV--------PTTHNKE-YHLLLY
MAST-4E-F08  ICLLA--GK-RDFRMIAFPWLLNLWTTIFGILSAIYWGP-LKHVCYGLAFVAAGSLYVELIRISGTKNVKSL-----PMFP-----ERKRLLQF
MAST-4E-A11  ICLLA--GK-RDFRMIAFPWLLNLWTTIFGILSAIYWGP-LKHVCYGLAFVAAGSLYVELIRISGTKNVKSL-----PMFP-----ERKRLLQF
MAST-4E-A02  VTIMTETGKTPDHNLILQLMLGITWIELFGFMGNLYDGF-FKDVCIALACCGMLVLYYAIFQ-TWTNNKD----------TLDRLKQRYELVVL
MAST-4E-C05  VTIMTETGKTPDHNLILQLMLGITWIELFGFMGNLYDGF-FKDVCIALACCGMLGLYYAIFQ-TWTNNKD----------TLDRLKQRYELVVL
MAST-7B-G22  RVVHSEEPTG-DF--TTKTIMCFIFTNVMGVTAAIQAEPGAKWAFFLAGIAGAAKLLHFFYVAWSE-EVQH-----MEQESLS---ARYQLMIL

MerMAID 1    LMVSWIVFPILYIVGPECFGLISNQFSIIGHVIGDVISKNLWGLMAWKLRLKIKDGQPM
MerMAID 2    FMTSWLVFPLMFILGPEMLNVMPFEWTLVGHCIGDLISKNAFGMLAWQYTKYLHRDEIK
MerMAID 3    LMVSWILFPILYIVGPECFGLISNQFSIIGHVIGDIISKNLWGLMAWKLRLKIKDGQPM
MerMAID 4    LMVSWILFPILYIVGPECFGLISNQFSIIGHVIGDIISKNLWGLMAWKLRLKIKDGQPM
MerMAID 5    LMVSWILFPILYIVGPECFGLISNQFSIIGHVIGDVISKNLMGLMAWKLRLKIKDGQPM
MerMAID 6    FMASWLVFPIMFIMGPEMTGIIPFEYTLVGHCVGDLISKNAFGILAWQYTLHIFKQ--K
MerMAID 7    FMGSWLVFPILFIVGPEMTNLVAFELTLVGHCVGDLIAKNAFGLLAWQYTKYVYKKNLK
MAST-4E-F08  LMITWLIFPVLFSLGPEYGAVITFRQSALFHAIGDLLSKNLMGFFSWSLGNTMLDRLER
MAST-4E-A11  LMITWLI----------------------------------------------------
MAST-4E-A02  LLVSWLLFPAFYILGPNMMKIVNNQISVIGHVVGDLLAKNIWGMLAWKFNRKTSS--S-
MAST-4E-C05  LLVSWLLFPAFYILGPNMMKIVNNQISVIGHVVGDLLAKNIWGMLAWKFNRKTSS--SS
MAST-7B-G22  MCLTWTTFPLVFLLGPEMTYILRYEGSAISFAVLDLLCKNLFGFMCWHFNFRNAW--S-
```
