# Supplementary material for: Comparative genomics reveals new functional insights in uncultured MAST species
Source: ISME J. 2021 Jan 15;15(6):1767–81. doi: 10.1038/s41396-020-00885-8 (PMC8163842; doi:10.1038/s41396-020-00885-8)
Supplement: Supplementary file 9 — Figure S8 [file 41396_2020_885_MOESM9_ESM.pdf]

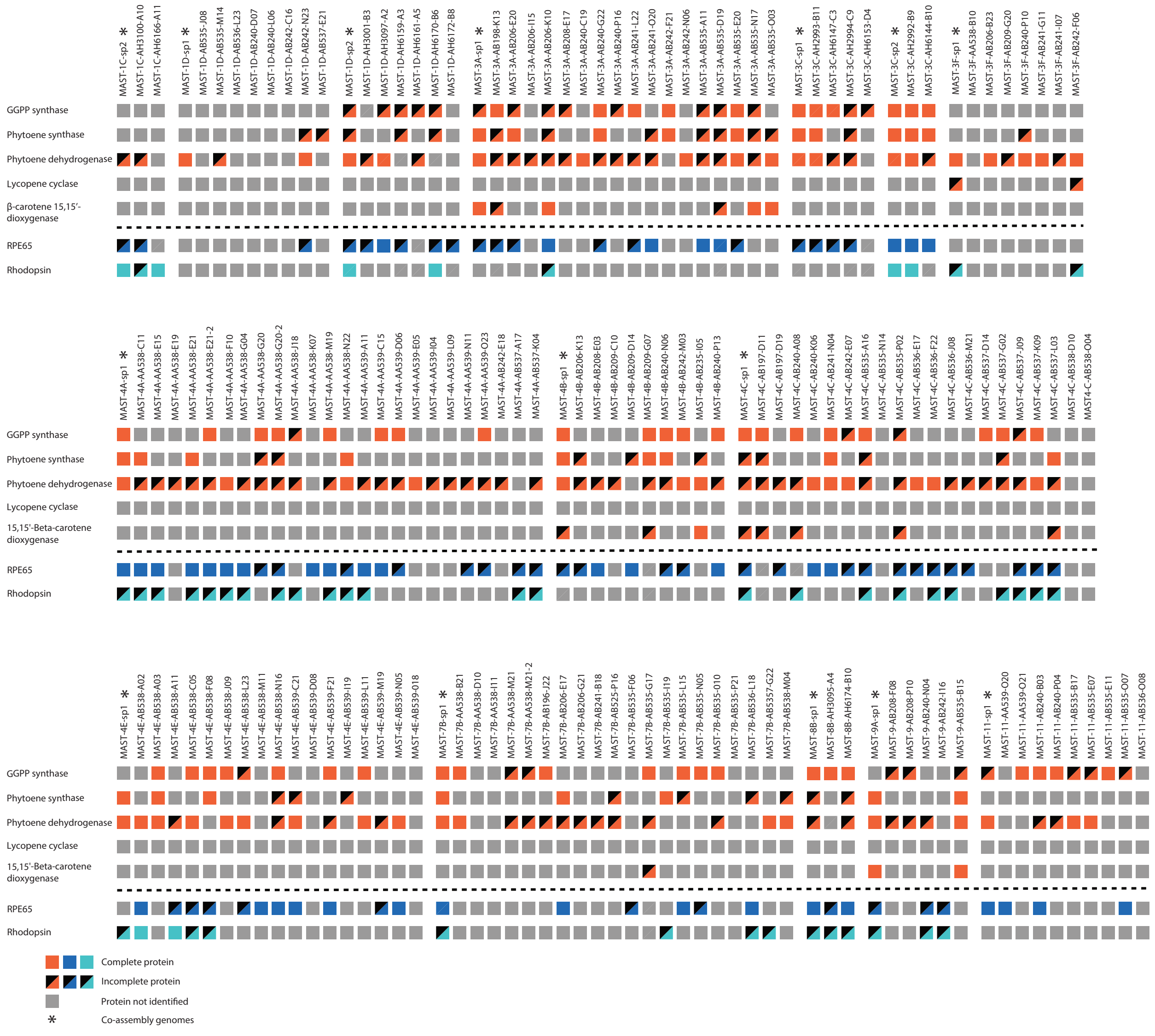

MAST-1C-sp2
MAST-1C-AH3100-A10
MAST-1C-AH6166-A11
MAST-1D-sp1
MAST-1D-AB535-J08
MAST-1D-AB535-M14
MAST-1D-AB536-L23
MAST-1D-AB240-D07
MAST-1D-AB240-L06
MAST-1D-AB242-C16
MAST-1D-AB242-N23
MAST-1D-AB537-E21
MAST-1D-sp2
MAST-1D-AH3001-B3
MAST-1D-AH3097-A2
MAST-1D-AH6159-A3
MAST-1D-AH6161-A5
MAST-1D-AH6170-B6
MAST-1D-AH6172-B8
MAST-3A-sp1
MAST-3A-AB198-K13
MAST-3A-AB206-E20
MAST-3A-AB206-I15
MAST-3A-AB206-K10
MAST-3A-AB208-E17
MAST-3A-AB240-C19
MAST-3A-AB240-G22
MAST-3A-AB240-P16
MAST-3A-AB241-L22
MAST-3A-AB241-O20
MAST-3A-AB242-F21
MAST-3A-AB242-N06
MAST-3A-AB535-A11
MAST-3A-AB535-D19
MAST-3A-AB535-E20
MAST-3A-AB535-N17
MAST-3A-AB535-O03
MAST-3C-sp1
MAST-3C-AH2993-B11
MAST-3C-AH6147-C3
MAST-3C-AH2994-C9
MAST-3C-AH6153-D4
MAST-3C-sp2
MAST-3C-AH2992-B9
MAST-3C-AH6144-B10
MAST-3F-sp1
MAST-3F-AA538-B10
MAST-3F-AB206-B23
MAST-3F-AB209-G20
MAST-3F-AB240-P10
MAST-3F-AB241-G11
MAST-3F-AB241-I07
MAST-3F-AB242-F06
GGPP synthase
Phytoene synthase
Phytoene dehydrogenase
Lycopene cyclase
β-carotene 15,15’-dioxygenase
RPE65
Rhodopsin
MAST-4A-sp1
MAST-4A-AA538-C11
MAST-4A-AA538-E15
MAST-4A-AA538-E19
MAST-4A-AA538-E21
MAST-4A-AA538-E21-2
MAST-4A-AA538-F10
MAST-4A-AA538-G04
MAST-4A-AA538-G20
MAST-4A-AA538-G20-2
MAST-4A-AA538-J18
MAST-4A-AA538-K07
MAST-4A-AA538-M19
MAST-4A-AA538-N22
MAST-4A-AA539-A11
MAST-4A-AA539-C15
MAST-4A-AA539-D06
MAST-4A-AA539-E05
MAST-4A-AA539-I04
MAST-4A-AA539-L09
MAST-4A-AA539-N11
MAST-4A-AA539-O23
MAST-4A-AB242-E18
MAST-4A-AB537-A17
MAST-4A-AB537-K04
MAST-4B-sp1
MAST-4B-AB206-K13
MAST-4B-AB208-E03
MAST-4B-AB209-C10
MAST-4B-AB209-D14
MAST-4B-AB209-G07
MAST-4B-AB240-N06
MAST-4B-AB242-M03
MAST-4B-AB235-I05
MAST-4B-AB240-P13
MAST-4C-sp1
MAST-4C-AB197-D11
MAST-4C-AB197-D19
MAST-4C-AB240-A08
MAST-4C-AB240-K06
MAST-4C-AB241-N04
MAST-4C-AB242-E07
MAST-4C-AB535-A16
MAST-4C-AB535-N14
MAST-4C-AB535-P02
MAST-4C-AB536-E17
MAST-4C-AB536-F22
MAST-4C-AB536-J08
MAST-4C-AB536-M21
MAST-4C-AB537-D14
MAST-4C-AB537-G02
MAST-4C-AB537-J09
MAST-4C-AB537-K09
MAST-4C-AB537-L03
MAST-4C-AB538-D10
MAST4-C-AB538-O04
GGPP synthase
Phytoene synthase
Phytoene dehydrogenase
Lycopene cyclase
15,15'-Beta-carotene dioxygenase
RPE65
Rhodopsin
MAST-4E-sp1
MAST-4E-AB538-A02
MAST-4E-AB538-A03
MAST-4E-AB538-A11
MAST-4E-AB538-C05
MAST-4E-AB538-F08
MAST-4E-AB538-J09
MAST-4E-AB538-L23
MAST-4E-AB538-M11
MAST-4E-AB538-N16
MAST-4E-AB539-C21
MAST-4E-AB539-D08
MAST-4E-AB539-F21
MAST-4E-AB539-I19
MAST-4E-AB539-L11
MAST-4E-AB539-M19
MAST-4E-AB539-N05
MAST-4E-AB539-018
MAST-7B-sp1
MAST-7B-AA538-B21
MAST-7B-AA538-D10
MAST-7B-AA538-I11
MAST-7B-AA538-M21
MAST-7B-AA538-M21-2
MAST-7B-AB196-J22
MAST-7B-AB206-E17
MAST-7B-AB206-G21
MAST-7B-AB241-B18
MAST-7B-AB525-P16
MAST-7B-AB535-F06
MAST-7B-AB535-G17
MAST-7B-AB535-I19
MAST-7B-AB535-L15
MAST-7B-AB535-N05
MAST-7B-AB535-010
MAST-7B-AB535-P21
MAST-7B-AB536-L18
MAST-7B-AB5357-G22
MAST-7B-AB538-M04
MAST-8B-sp1
MAST-8B-AH3095-A4
MAST-8B-AH6174-B10
MAST-9A-sp1
MAST-9-AB208-F08
MAST-9-AB208-P10
MAST-9-AB240-N04
MAST-9-AB242-I16
MAST-9-AB535-B15
MAST-11-sp1
MAST-11-AA539-O20
MAST-11-AA539-O21
MAST-11-AB240-B03
MAST-11-AB240-P04
MAST-11-AB535-B17
MAST-11-AB535-E07
MAST-11-AB535-E11
MAST-11-AB535-O07
MAST-11-AB536-O08
GGPP synthase
Phytoene synthase
Phytoene dehydrogenase
Lycopene cyclase
15,15'-Beta-carotene dioxygenase
RPE65
Rhodopsin
Complete protein
Incomplete protein
Protein not identified
Co-assembly genomes
